# A meta-ethnography of the factors that shape link workers’ experiences of social prescribing

**DOI:** 10.1186/s12916-024-03478-w

**Published:** 2024-07-04

**Authors:** Amadea Turk, Stephanie Tierney, Bernie Hogan, Kamal R. Mahtani, Catherine Pope

**Affiliations:** 1 Nuffield Department of Primary Care Health Sciences, University of Oxford, Oxford, UK; 2 Oxford Internet Institute, University of Oxford, Oxford, UK

**Keywords:** Meta-ethnography, Qualitative research, Social prescribing, Link workers, Primary care

## Abstract

**Background:**

Social prescribing is gaining traction internationally. It is an approach which seeks to address non-medical and health-related social needs through taking a holistic person-centred and community-based approach. This involves connecting people with and supporting them to access groups and organisations within their local communities. It is hoped that social prescribing might improve health inequities and reduce reliance on healthcare services. In the UK, social prescribing link workers have become core parts of primary care teams. Despite growing literature on the implementation of social prescribing, to date there has been no synthesis that develops a theoretical understanding of the factors that shape link workers’ experiences of their role.

**Methods:**

We undertook a meta-ethnographic evidence synthesis of qualitative literature to develop a novel conceptual framework that explains how link workers experience their roles. We identified studies using a systematic search of key databases, Google alerts, and through scanning reference lists of included studies. We followed the eMERGe guidance when conducting and reporting this meta-ethnography.

**Results:**

Our synthesis included 21 studies and developed a “line of argument” or overarching conceptual framework which highlighted inherent and interacting tensions present at each of the levels that social prescribing operates. These tensions may arise from a mismatch between the policy logic of social prescribing and the material and structural reality, shaped by social, political, and economic forces, into which it is being implemented.

**Conclusions:**

The tensions highlighted in our review shape link workers’ experiences of their role. They may call into question the sustainability of social prescribing and the link worker role as currently implemented, as well as their ability to deliver desired outcomes such as reducing health inequities or healthcare service utilisation. Greater consideration should be given to how the link worker role is defined, deployed, and trained. Furthermore, thought should be given to ensuring that the infrastructure into which social prescribing is being implemented is sufficient to meet needs. Should social prescribing seek to improve outcomes for those experiencing social and economic disadvantage, it may be necessary for social prescribing models to allow for more intensive and longer-term modes of support.

**Supplementary Information:**

The online version contains supplementary material available at 10.1186/s12916-024-03478-w.

## Background

Social prescribing is an approach to health and wellbeing that seeks to acknowledge and address some of the effects of the social determinants of health [1]. While various definitions, models, and implementation approaches exist internationally, a recent consensus study defines social prescribing as “a means for trusted individuals in clinical and community settings to identify that a person has non-medical, health-related social needs and to subsequently connect them to non-clinical supports and services within the community by co-producing a social prescription—a non-medical prescription, to improve health and well-being and to strengthen community connections” [2] (p.9). In practice it entails working with service users (patients) to provide them with personalised support by co-producing an action plan that assesses their needs, strengths, and interests, and empowering them to take greater control of their health and wellbeing. Service users are then supported to access community resources [2]. Examples may include referrals to gyms and other lifestyle support groups and services, arts, and cultural activities [3], green spaces [4], as well as services that can offer support with housing, finances, and welfare advice [5]. Social prescribing is gaining traction internationally, with at least 25 countries around the world introducing it as of 2023 [1].

Social prescribing is frequently framed as a way of addressing health inequities by responding to the social determinants of health [6], with policy makers stating that it is “effective at targeting the causes of health inequalities” [7]. Social prescribing is presented as a “community-centred” approach to health and wellbeing, which “seeks to draw on and strengthen community capacity to take collective action that will in turn lead to changes in health or the social determinants of health” [8] (p.19)*.* It has also been described as an “asset-based” approach, which aims to recognise and focus on a community’s strengths (rather than its deficits) as a foundation for creating social change [8].

In England, social prescribing is now a key part of the National Health Service Long-Term (NHS) Plan and Personalised Care Agenda [9], with hopes that it will reduce the reliance on NHS primary care services [10] as well as reduce clinician workload [5]. Its delivery is supported through the introduction of social prescribing link workers, who have been brought on as new non-clinical members of the NHS primary care workforce [11]. Link workers support service users in identifying issues affecting their health and wellbeing, and to access community-based support. A key aspect of the link worker role is to undertake activities aligned with an “asset-based” approach. This involves working collaboratively with partners from across the health and care system to both identify and fill gaps in community services provision and encourage community mobilisation [5, 12].

The ways in which link workers operate locally can vary considerably [13], with some providing intensive open-ended support, while others operate within clearly defined boundaries providing “light touch” [14] support which may be limited to signposting [6]. Currently, in England, link workers can be employed directly through funds made available to Primary Care Networks (PCNs) or contracted through third-sector organisations [13]. The NHS Long Term Workforce Plan, which lays out a cost-effective approach to meeting current and future healthcare demands, commits to increasing the number of link workers employed from 3000 in September 2022 to 9000 by 2036/37 [15].

The literature on social prescribing and link workers is growing rapidly, both across the UK and internationally. Many of these studies are qualitative explorations of the experiences, implementation, and effects of social prescribing in different settings. A consistent theme from current research has been the need to develop a refined theoretical understanding of processes that shape the ways in which link workers undertake their role [16, 17]. However, to date there has been no synthesis that attempts to develop a theoretical understanding of the factors that shape link workers’ experiences of their role.

## Aim and rationale

The aim of this review was to synthesise existing qualitative research on factors shaping link workers’ experiences of their role and the ways in which the role is being implemented. Our synthesis sought to develop a novel conceptual framework which explains how social prescribing is being enacted and experienced by frontline social prescribing staff. The review aimed to answer the following research questions:

1. What factors shape experiences and perceptions of link workers carrying out social prescribing?
2. What factors influence the ways in which link workers work with service users?

## Methods

### Study design

As the aim of this review was to develop a novel theoretical understanding of the factors that shape link workers’ experiences of social prescribing, we employed a meta-ethnographic approach to evidence synthesis to integrate qualitative literature on the link worker role. Meta-ethnography [18] is a theory-generating and interpretive methodology for the synthesis of qualitative evidence [19]. The approach is interpretive, rather than aggregative, and involves systematically comparing and translating data from qualitative studies such as participant quotes (first-order constructs) and concepts/themes developed by authors of a primary study (second-order constructs) [19]. This process then enables the identification and development of new overarching theories and conceptual models (sometimes referred to as third-order constructs) [19, 20] through reinterpretation (re-analysis) of published findings [21]. Conducting a meta-ethnography involves seven stages: getting started, deciding what is relevant to the initial interest, reading the studies, determining how the studies are related, translating the studies into one another, synthesising the translations, and expressing the synthesis. The protocol for the review was registered on PROSPERO [CRD42021264595]. We followed the eMERGe guidance when conducting and reporting this meta-ethnography [19].

### Identification of studies: deciding what is relevant

We conducted a systematic search of key electronic databases covering a mixture of health and social science literature to identify published qualitative studies containing data about factors that shape link workers’ experiences of their role in social prescribing and its implementation. We searched nine databases in August 2021 with the help of an information specialist (NR). The databases searched were Medline (OvidSP) [1946–present], Embase (OvidSP) [1974–present], CINAHL (EBSCOHost) [1982–present], PsycINFO (OvidSP) [1806–present], Health Management Information Consortium (HMIC) (OvidSP) [1979–July 2021], Social Science Citation Index (Web of Science Core Collection) [1900–present], and Sociology Collection—Applied Social Sciences Index & Abstracts, Sociological Abstracts and Sociology Database (Proquest) [1952–present].

The search strategy consisted of title/abstract keywords and subject headings describing the link worker role, primary care, and qualitative research. The search terms recognised the fact that link workers are sometimes referred to by other titles such as social prescriber or community connector [13]. No date, language, or publication type limits were applied to the search. Full search strategies are available in Additional File 1. In addition, we searched OpenGrey for grey literature such as policy reports. As the literature on social prescribing is growing rapidly, we set up a Google Scholar alert with the term “social prescribing” to keep abreast of newly published papers. This continued until the point of finalising our analysis in November 2023. We also scanned the reference lists of included papers to identify other potentially eligible studies that may have been missed by our searches. The inclusion criteria for studies in our review focused on link worker models of social prescribing and can be found in Table 1.

**Table 1:** Criteria for publications to be included in the review

Inclusion	Exclusion
• Literature from any country engaging in social prescribing with a link worker model • Qualitative studies (that used a qualitative approach to data collection and analysis) • Mixed methods studies where qualitative components could be distinguished from the quantitative ones • Studies with perspectives from link workers employed through primary care as well as other agencies • Studies with perspectives from services users, primary care staff, or voluntary and community sector staff which discussed factors relevant to link workers’ experiences of their roles • Written in English • Grey literature with sufficient detail on how qualitative components were undertaken	• Studies not containing data related to the factors relevant to link workers’ experiences of their roles in social prescribing • Questionnaires/surveys with open-ended questions • Reviews • Conference abstracts • Theses • Studies that did not contain sufficient detail on how qualitative components were undertaken

The first author (AT) conducted the initial screening of the retrieved references by title and abstract to exclude irrelevant studies, and a sample (20%) was screened by a second reviewer to ensure that inclusion criteria were being applied consistently. Disagreements were resolved through discussion. Remaining texts were read in full and assessed for their relevance. All screening was managed using Rayyan, a systematic review management platform. The reference list of each included paper was scanned to identify further eligible papers. The same process and criteria were also applied to studies identified by the Google Scholar alerts.

### Quality appraisal

There is considerable debate on whether qualitative research should be subject to critical appraisal. Some argue that doing so stifles the interpretive and creative aspects of qualitative research, reducing it to a list of overly prescriptive technical procedures [22], or that the checklists do not appreciate the heterogeneity of approaches to qualitative research [23]. Noblit and Hare [18] did not advocate for formal appraisal of studies as part of meta-ethnography, as they argued that study quality would become apparent by how much it contributed to the synthesis [18, 24]. However, critical appraisal has been performed in meta-ethnographies to examine the overall quality of included papers and to identify gaps in the reporting [25, 26]. With this in mind, we undertook a critical appraisal of studies included in the review using the Critical Appraisal Skills Programme (CASP) Qualitative Studies Checklist [27], but did not exclude studies based on their scoring.

### Synthesis

Each stage of our synthesis was an iterative process during which the research team met regularly to discuss emerging ideas. These were also discussed with a patient and public involvement group and a stakeholder group which included social prescribing link workers, policy makers, and social prescribing managers (staff responsible for the delivery and oversight of social prescribing programmes).

### Reading the studies

All included publications were read and re-read by the lead author (AT) to identify and become familiar with their key concepts and to determine how studies related to one another by comparing their aims, focus, characteristics, theoretical approach, and findings. We extracted themes from each of the papers (second-order constructs) using a table in which these were noted along with illustrative data (first-order constructs). We also noted additional ideas that arose while reading the papers to help inform the development of third-order constructs.

### Determining how the studies are related

During this stage, we considered the relationships between the key concepts (second-order constructs) we extracted from the papers. This involved looking across the studies for similar or recurring concepts. We grouped them into initial categories, which we labelled using terminology that adequately described all the relevant concepts they contained. These were then juxtaposed and compared against each other to explore the potential relationships between the key concepts from the original studies. We drew initial “conceptual maps” (see Additional File 2) to aid the exploration of how these initial categories might relate to each other.

### Translating studies into one another

“Translating” is central to meta-ethnography. It describes the idea that each author is using their own “interpretive language” and therefore a comparison of conceptual terms across studies is needed. This comparison involves reading and understanding the meaning of the original authors’ interpretations, but no further conceptual development [26]. Second-order constructs from the included studies were compared systematically to identify the range of concepts and whether their meanings were similar or contradictory. This involved noting and summarising each second-order construct and its definition in an Excel table and mapping the presence of each construct across each of the included studies. During this process, we re-examined the initial categories developed in the previous stage and reorganised them based on the emerging translations.

### Synthesising translations

We undertook a reciprocal synthesis where the different studies included were viewed as a whole and from which we were then able to build a line of argument or overarching model [28], which explains the new emerging storyline of the synthesis [29]. We revisited the categories developed in previous stages through further discussion. This facilitated additional interpretation and allowed us to develop key overarching concepts (third-order constructs)—see Table 3. These final third-order constructs were then linked through additional discussion and analysis to develop a “line-of-argument synthesis”, which provides a further interpretation that puts any similarities and differences across studies into a new interpretive context [19]. This allowed us to produce an overarching conceptual framework of the experiences of link workers carrying out social prescribing.

### Findings

**Fig. 1:**
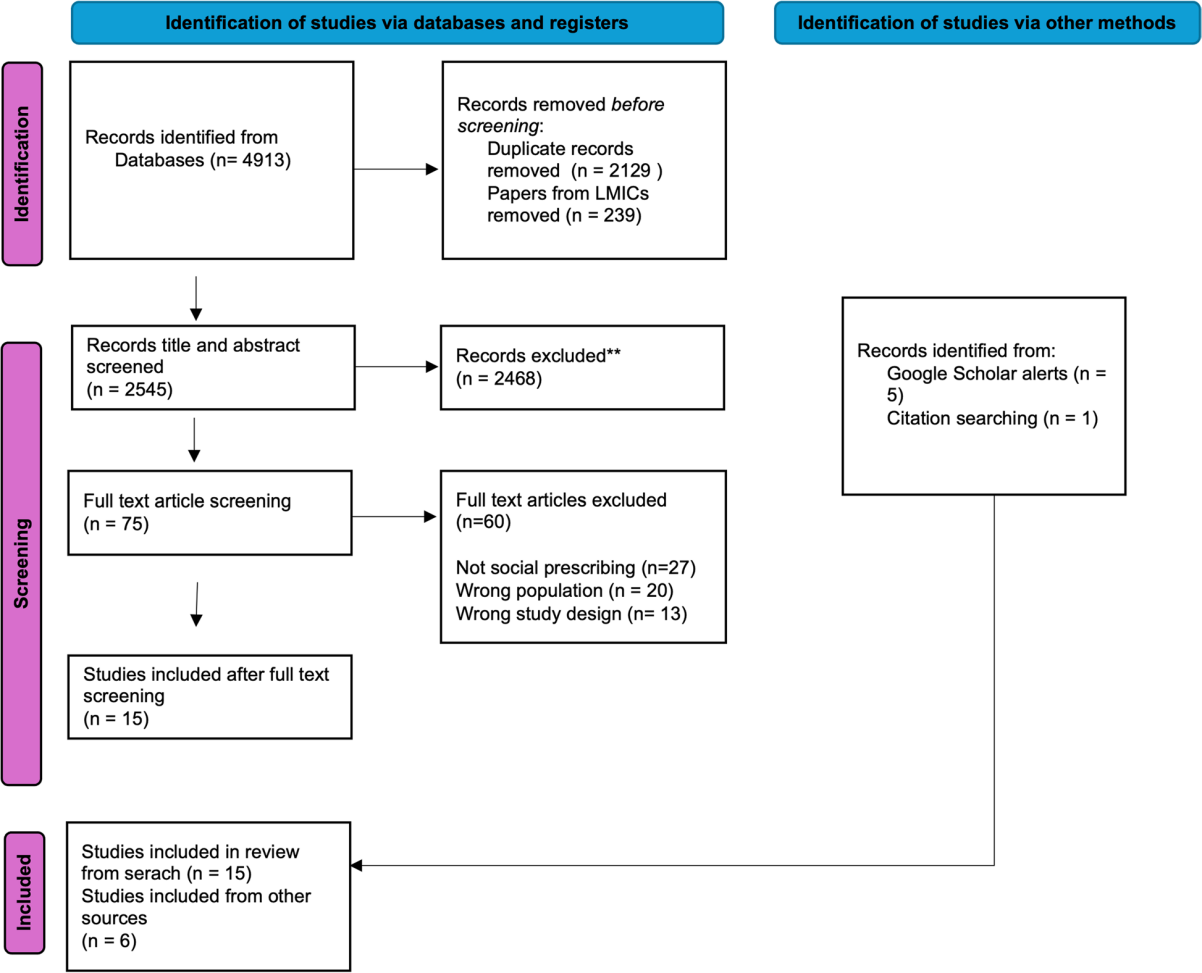
Flow diagram showing study selection

The searches of online databases yielded 2545 records. We screened each record’s title and abstract for relevance, after which we read 75 records at full text level. From the database search, 15 papers were included in the review, 5 additional papers were identified through the Google Scholar alerts, and a further paper was identified through reviewing the reference lists of included papers (see Fig. 1). In total, we included 21 studies published between 2017 and 2023, reporting the results of 17 different studies (5 publications report the findings from one larger project) (Table 2).

**Table 2:** Table of study characteristics. The * beside 6 of the publications indicates that they report findings from the same larger project

Authors	Aim	Country/ setting	Social prescribing service	Sample size	Sample characteristics	Method of data collection	Analysis
Beardmore 2019 [30]	To explore routes into social prescribing, and link worker’s experiences of the sector and potential career progression	urban/suburban area of South West England	Various services, details not provided	8	Members of the social prescribing workforce with varying levels of responsibility from six different organisations (NHS, Local Authority and voluntary and community sector (VCS))	Semi-structured interviews	Thematic analysis
Chng et al. 2021 [31]	To explore the implementation process of social prescribing in practice	Deprived areas of Scotland	Each intervention practice had a full-time salaried community links practitioner (CLP) appointed, who was employed by a Scottish Government-funded third-sector organisation. Intervention practices were also offered additional programme management support by the CLPs’ employing organisation	> 31 (exact number is unclear)	Practice staff with responsibility for leading the social prescribing programme, including link workers, as well as other key social prescribing stakeholders	Focus groups, email surveys, in-depth interviews	Thematic analysis
Fixsen et al. 2020 [32]	To critically examine a developing rural social prescribing scheme from multiple stakeholder perspectives and to present a relational model for local social prescribing schemes	Rural area, Shropshire, United Kingdom	A social prescribing initiative conducted in the rural area of Shropshire which forms part of the wider local health and wellbeing strategy to promote health and reduce health inequalities	24	Key stakeholders (practice managers, disability and employment advisors, social care workers, GPs, link workers, public health consultants, service users)	Interviews	Modified grounded theory
Fixsen et al. 2021 [33]	To qualitatively examine and compare the responses of three social prescribing schemes in Scotland to the COVID-19 pandemic	Deprived area Glasgow, Scotland	Social prescribing scheme delivered through the Scottish government where link worker support patients navigating support in the community	23	Link workers, GPs, social prescribing managers, representatives from the voluntary and community sector	Interviews	Inductive thematic approach
Frostick and Bertotti 2019 [34]	To identify the training, skills and experience social prescribing Link Workers, working with patients presenting with long-term conditions, need to carry out their role safely and effectively within primary care services	London, United Kingdom	Various—not described in detail	13	Link workers in post for 6 months or more- from three London-based social prescribing schemes	Interviews and focus groups	Thematic analysis
Griffith et al. 2022 [35]*	To identify factors shaping delivery context and link worker practices through the interacting logics of choice and care. To examine how these practices resonated with contemporary social prescribing discourses and the stated aims and objectives of national policy	Socioeconomically deprived urban area of North East England	A social prescribing scheme for those aged 40–74 with certain long-term conditions. Link workers were provided by two third-sector organisations one of which focused on community development and had a longstanding commitment to behaviour change. The scheme was partially funded through a Social Impact Bond	not clear	20 link workers; 13 interviews with service managers	Ethnographic fieldwork (interviews, focus groups, observation)	Thematic content analysis
Griffiths et al. 2023 [36]	To explore the perspectives of social prescribing staff in United Kingdom (UK) NHS social prescribing services	Central England	Social prescribing services delivered across a county in central England	18	Link workers	Interviews	Thematic analysis
Hazeldine et al. 2021 [37]	To investigate the early implementation of link worker social prescribing using a researcher in residence model	South West England, United Kingdom	Two social prescribing services The first service was delivered by consortium of six Voluntary and Community sector partners, with objectives to develop social capital and the wellbeing of individuals and communities, covering an adult population of 221,673. Link workers were based in a GP practice The second service was based in a single GP practice where they shared an office and had regular contact with colleagues. The service covered a population of 46,000. Link workers were employed by a single Community Development Trust	13	11 link workers and 2 social prescribing managers	Structured conversations guided by a topic guide. Researcher made detailed field notes	Thematic analysis
Holding et al. 2020 [38]	To understand the challenges of delivering support and the resources and community infrastructure required for successful delivery of social prescribing	England	A national social prescribing programme supporting people at risk of, or experiencing, loneliness to connect with their communities. It involved link workers and volunteers working closely with service users for up to 12 weeks to develop social links through signposting to community activities and other support	24	9 volunteers and 15 link workers	Semi-structured interviews	Interpretive thematic analysis
Khan et al. 2021 [39]	To understand perceptions of social prescribing within the wider community	Four socioeconomically disadvantaged areas in North-West England	N/A	37	Members of the public	Focus groups and interviews	Thematic analysis
Mackenzie et al. 2020 [40]	To explore the concept of “fantasy paradigms” empirically using data from an evaluation of a social prescribing case	Glasgow, Scotland	Community Links Practitioners (CLPs) employed as an additional resource within practices: to engage with ‘vulnerable’ patients and to connect them with community resources. The CLPs acted as a channel between GP practice and community services; and to foster change within General Practice	47	Professionals involved with delivering social prescribing	Interviews	Thematic analysis
Moffat et al. 2017 [41]*	To describe the experiences of patients with long-term conditions who are referred to and engage with a Link Worker social prescribing programme and identify the impact of the Link Worker programme on health and wellbeing	West Newcastle upon Tyne, United Kingdom	Social prescribing link workers trained in behaviour changed methods who offer a holistic and personalised service. They receive referrals from primary care health professionals and connect patients to community organisations. Patients can participate in the programme for up to 2 years, with the option for Link Worker discretion for extended support if necessary. Contact frequency is mutually agreed upon and varies based on individual needs. Meetings with link workers can occur face-to-face or through various digital communication channels	30	Adults with long-term conditions	Interviews	Thematic analysis
Moore et al. 2023 [42]	To gain an understanding of the experiences of social prescribing link workers regarding their professional identities within their roles	Clinical Commissioning Group area covering a range of city, urban, and rural environments, although it was more rural and less ethnically or socioeconomically diverse than the national average	Mixture of link workers employed directly by the Clinical Commissioning Group or commissioned through a social prescribing provider	13	Social prescribing link workers	Interviews	Thematic analysis
Morris et al. 2023 [43]*	To explore accounts of how an existing social prescribing service adapted to meet clients’ needs in the first wave of the pandemic, and of how clients experienced these changes	Deprived urban area of North East England	A social prescribing scheme for those aged 40–74 with certain long-term conditions. Link workers were provided by two third-sector organisations and could support patients for up to approximately 3.5 years	57	Clients (*n* = 44), link workers (*n* = 5) and service provider managerial staff (*n* = 8)	Remote interviews	Thematic analysis
Pollard et al. 2023 [44]*	To explore how a social prescribing intervention was delivered by link workers and the experiences of those referred to the intervention	Economically deprived urban area of the North of England	A social prescribing scheme for those aged 40–74 with certain long-term conditions. On referral from GP practices, patients were assigned a link worker. The link worker was expected to support patients to access relevant local community services, or in some cases, to support them to develop self-directed programmes. Link workers support patients for an average of 18 months, but in some cases this support was extended up to four years. Link workers were provided by two third-sector organisations	39	20 link workers; 19 service users	Participant observation, focus groups, interviews	Thematic analysis
Rhodes and Bell 2021 [45]	To explore the challenges of working as a social prescriber and experiences of training and support received	Greater London, United Kingdom	Various—not described in detail	9	Social prescribers	Interviews	Thematic analysis
Simpson et al. 2020 [46]	To explore the application of social prescribing with people living with motor neurone disease (MND)	North West Coast of England	Link workers focused MND, but social prescribing model not described in detail	13	4 link workers, 9 patients with MND	Interviews	Thematic analysis
Skivington et al. 2018 [16]	To investigate issues relevant to implementing a social prescribing programme to improve inter-sectoral working to achieve public health goals	Scotland	Community links practitioners (CLPs) with a third-sector or community development background and were employed to work in GP practices. Referrals were received from primary care professionals, to support patients to access community organisations, with the aim of improving their health and wellbeing, and mitigating the negative impacts of the social determinants of health	36	6 link workers and 30 community organisation representatives	Interviews	Thematic analysis
White et al. 2022 [47]	To evaluate from multiple perspectives the effectiveness of one city-based social prescribing service	Socioeconomically disadvantaged urban area of Northern England	A social prescribing service delivered by two community organisations and available to those aged 16 years and over. Support was provided by Link Workers based within GP practices and other locations including community centres	57	Link workers, GPs, social workers, patients	Interviews, focus groups, surveys	Thematic analysis
Wildman et al. 2019 [48]*	To explore link workers’ definitions of their role and the skills and qualities they perceive to be essential for effective client engagement	Socioeconomically deprived areas in North East England	The social prescribing service was delivered by four third‐sector organisations who employed link workers and received referrals from primary‐care practitioners based in 17 general practices	15	Link workers	Focus groups and interviews	Thematic analysis
Wildman et al. 2019 [49]*	To explore experiences of social prescribing among people with long-term conditions one to two years after their initial engagement with a social prescribing service	Socioeconomically disadvantaged area of North East England	A social prescribing scheme for those aged 40–74 with long-term conditions. Link workers provided by two third-sector organisations. Link workers were trained in behaviour change methods. These techniques emphasised service users' choice and control over their decisions and behaviours	24	Service users	Semi-structured follow-up interviews	Grounded theory approach, thematic analysis

### Study characteristics

The 21 studies included in the meta-ethnography (Table 2) were published between 2017 and 2023. They included over 531 participants and a range of social prescribing initiatives. Sample sizes varied between 8 and to more than 31 participants; in one of the studies, it was unclear exactly how many participants took part [31]. Seven studies included perspectives exclusively from link workers or social prescribing managers; seven included the perspectives of link workers in addition to other staff (e.g. GPs or voluntary and community sector staff); two included the perspectives of link workers and patients; two included a mixture of perspectives from link workers, other staff, and patients; two discussed service user perspectives only; and one study included perspectives from members of the public.

Seventeen of the studies were based in England, and the remaining four in Scotland. While our inclusion criteria did allow for studies outside of the UK, none of the international publications met the criteria. Eight studies explicitly stated that they were based in socioeconomically disadvantaged areas.

Most of the studies employed interview techniques (either in person or online), some used focus groups, while others used a mixture of techniques such as interviews, focus groups, participant observation, and ethnographic fieldwork. None of the grey literature identified through the searches provided enough detail on data collection or analysis to be included in the review.

### Critical appraisal

Following the CASP Checklist [50], most of the studies included clear and relevant research aims, used appropriate methodologies and research designs, and clearly stated their findings (see Additional File 3). However, some did not explicitly discuss reflexivity, or ethical considerations beyond indicating that the studies had received approval from research ethics committees.

### Synthesis

Through the synthesis we developed a “line of argument”, whereby a narrative is produced based on third-order constructs [18]. Our analysis highlights inherent tensions present at each of the levels that social prescribing operates at, arising from a mismatch between the policy logic of the intervention and the material and structural reality into which social prescribing is being implemented. These tensions shape link workers’ experiences of their role and could jeopardise social prescribing’s sustainability.

The narrative is presented in the proceeding sections, with the third-order constructs organised under four headings that represent each of the levels at which social prescribing operates—the link worker level, the organisational level, the wider system level, and the patient level. Tensions present at each level are outlined.

Throughout the next section, authors’ interpretations (from included papers) are identified using quotation marks, while direct quotations from study participants in the included studies are presented in italics with quotation marks. Table 3 presents the third-order constructs and how they fit into our overall line of argument and indicates which papers made contributions to each third-order construct.

**Table 3:** Formation of third-order constructs from second-order constructs in each included paper. Publications with * were based on the same larger study. The “X” indicates to which parts of the line of argument each paper contributed

**Third-order constructs**	Role definition as a double-edged sword	The quasi-professional status of the link worker role	Balancing tensions in the relational nature of link working	A reliance on individual characteristics as drivers of success	Organisational buy-in	The pressure of targets	Support for Link workers	A struggling wider health and care system	Local variation and structural poverty	Link workers as an additional resource	Challenges to the dominant discourses of “choice” and “empowerment”	The temporal and material requirements for person-centred care
**Contribution to line of argument**	Tensions in the link worker role				Tensions at the organisational level			Tensions at the wider system level			Tensions at the service-user level	
Beardmore 2019 [30]	X	X	X	X	X		X	X				
Chng et al. 2021 [31]			X									
Fixsen et al. 2020 [32]							X	X				X
Fixsen et al. 2021 [33]	X	X		X	X			X				
Frostick and Bertotti 2019 [34]	X		X	X								
Griffith et al. 2022 [35]*	X		X			X		X	X		X	X
Griffiths et al. 2023 [36]	X	X	X				X	X			X	
Hazeldine et al. 2021 [37]	X			X			X	X	X	X		
Holding et al. 2020 [38]	X			X				X	X			
Khan et al. 2021 [39]				X				X				
Mackenzie et al. 2020 [40]										X	X	X
Moffat et al. 2017 [41]*			X	X								X
Moore et al. 2023 [42]	X	X			X						X	
Morris et al. 2023 [43]*				X								
Pollard et al. 2023 [44]*	X				X	X		X				X
Rhodes and Bell 2021 [45]	X	X					X					
Simpson et al. 2020 [46]		X	X					X	X			
Skivington et al. 2018 [16]			X	X				X		X		X
White et al. 2022 [47]	X		X		X					X	X	X
Wildman et al. 2019 [48]*		X	X	X	X	X				X		X
Wildman et al. 2019 [49]*			X						X			X

### The link worker level

Our analysis revealed tensions relating to the ways in which the link worker role itself has been implemented into the health and care system. These tensions relate to role definition, professional identity, relational working, and the reliance on individual link worker characteristics as drivers of success. Each of these tensions has implications for link workers’ workloads, and the ways in which their roles are understood and accepted by others working in healthcare. These factors may act as stressors on the link worker role, threatening retention and in turn risking the ability for social prescribing to deliver desired outcomes.

#### Role definition as a double-edged sword

Link workers have been introduced into the health and care system as a new role, often with a flexible remit and without a clear job description [33, 34, 45]. On the one hand, this flexibility could allow link workers to tailor the way they worked to fit with the population and area they serve, ultimately helping them to provide person-centred care [36, 42]. However, this same lack of clear role definition could cause role stress through workload overload and complexity. It could also lead to confusion/ambivalence among other members of staff about what social prescribing is and what the link worker role could offer, in turn contributing to inappropriate referrals.

The flexible nature of the link worker role meant it had been operationalised in different ways across different organisations [33], with “their job scope and remit being poorly defined from the outset” and their role being “not well understood by external referrers” [45] (p.3). This could lead to unrealistic expectations or confusion around what is achievable through social prescribing [47] and to the referral of patients with complex needs that exceeded the remit of the link worker role [33, 36, 37].

Link workers were sometimes used to “fill gaps”, which is an identified risk of having unclear professional identities in newer professions [42]. In the studies, parameters of the link worker role were frequently exceeded due to concerns that nobody would support patients otherwise, because “no other professions would deviate from their role boundaries” [42] (p.5). Furthermore, a lack of understanding of referral criteria could lead to link workers feeling that social prescribing was being “used as a dumping ground for difficult patients” [34] (p.6)*.* The filling of gaps was particularly pertinent in the context of overstretched and underfunded statutory and mental health services. In these contexts, link workers often ended up taking on more clinical risk [38, 42] or providing more specialist support such as acting as unqualified social or advocacy workers [38], due to long waiting lists or lack of availability for more specialised services. Not having onward referral options may also limit link workers’ ability to set boundaries with service users [16, 48].

#### The quasi-professional status of the link worker role

Link workers have been introduced into the health and care system as a new “professional” role, but without the formal training and registration that would afford them professional status [33] and identity to give them the confidence needed to deal with complex cases they encounter in their work [42, 45]. Coupled with the lack of understanding or ambivalence about the role from other primary care professionals, link workers sometimes felt that they were “treated as outsiders going apparently unrecognised by practice staff” [44] and sometimes found it difficult to become integrated and visible in primary care due to “entrenched professional hierarchies” [35].

Formal training is considered essential for the building of professional identity and status [42]; however, across the studies there was variation in the training link workers received. While formal training could increase link worker confidence in performing their role [48], some link workers reported that it was often minimal, inconsistent and non-standardised [42]. Some needed to draw on skills and training they received in previous roles [42] and those taking on more senior responsibilities felt like they did not have enough training for this [30]. In other contexts link workers reflected that the training they had received was overly theoretical and lacked practical elements to prepare them for dealing with the range, severity and complexity of issues they encountered [45, 48]; or for dealing with specific health conditions [46]. Furthermore, opportunities for career progression within the role were often limited, which could affect role retention [30, 33]. However, it was acknowledged that formal training available to link workers was improving and that this may suggest social prescribing is moving towards integration into mainstream practice [42].

The absence of a shared and commonly understood role and professional identity and pathway could threaten professional resilience and fulfilment [42] and the retention of link workers [30, 42]. However, it was noted that professionalising the role, by bringing in formal qualifications and registration, may have consequences that hinder the successful delivery of social prescribing [42]. On the one hand, formal professionalisation and registration may increase the legitimacy of the role, as well as improve equitable access to social prescribing and consistency for patients through greater standardisation [42]. Yet it was noted that making the link worker role a registered profession may make it more restricted and remove individuality and the flexibility currently available in social prescribing, which may be key to delivering to person-centred care [36, 42]. It may also present a barrier to connecting with service users who may find it easier to relate to someone who is not a “professional” and “more like them” [42]*.* Finally, creating a “barrier to entry” through requiring registration and specific training may impact recruitment to the role which was already difficult [42].

#### Balancing tensions in the relational nature of link working

Social prescribing hinges on link workers establishing and nurturing relationships between individuals and organisations in a way that builds trust and fosters collaboration [33–36, 41, 43, 47–49]. Relationships with link workers were sometimes likened to friendships by patients, and contrasted to interactions they were used to having with healthcare professionals—often depicted as rushed and impersonal [49]. Link workers were described as a “companion” with whom patients could share their stories; these close relationships were necessary for the success of social prescribing [49]. This relational nature of social prescribing work was described as being “a bit of a balancing act” between being a “friend but not a friend” [48] (p.996), where it was also vital to maintain boundaries in a way that did not foster patient dependency [36] and protected link workers’ time and headspace [47]. A key strategy for setting boundaries with service users involved referrals into other agencies, although as described later, local community infrastructure may not always be readily available to permit this to happen [48].

Being able to get to know patients, to explore and understand their individual contexts, and “empowering” [34](p.6) them to make decisions for their health and wellbeing could be a “particularly satisfying” aspect of the link worker role [34] (p.6). A link worker in the study by Beardmore noted that “I just love working with people. It’s quite a privilege to be part of someone’s journey” [30] (p.44). The author noted that despite the potentially negative impact of the lower salary associated with the link worker role, link workers found satisfaction in the relational nature of their work and in supporting people [30]. However, in the study by Moore et al., link workers felt that they were not being remunerated enough for the workload and risk they often found themselves taking on [42].

Link workers’ ability to form relationships with other healthcare professionals and community organisations was another key factor to the success of social prescribing services [16, 31, 34, 36, 48]. They were uniquely placed to act as a bridge between different stakeholders due to their understanding of both primary care and the voluntary and community sector [16]. This networking aspect of the role required the link worker and social prescribing service to be fully embedded into the practice and that when it was, it could lead to the whole practice developing links with community organisations [31]. However, in other cases it could be challenging to transition from individual relationships between staff to more enduring collaborations with organisations, irrespective of the individuals involved [16]. Staff continuity is a concern for the longevity of these collaborative relationships, which can be compromised if link workers leave their role due to poor job satisfaction.

#### A reliance on individual characteristics as drivers of success

As described above, we identified no singular, clear definition or remit of the link worker role, or specific previous experience or training required to undertake it. However, it was apparent that the skills, experience and personal characteristics that link workers possessed were often the driving force behind social prescribing programmes, meaning the role should not be undertaken by “just anyone” [39] (p.4).

Previous professional backgrounds often influenced how link workers approached their role and the amount of confidence they had in its execution [38]. For example, link workers who previously worked in mental health professions or had mental health training felt more confident and able to make decisions [37].

Empathy, being non-judgemental and supportive, compassionate, having listening skills, and being able to put people at ease were all considered essential traits for link workers [16, 32, 34, 39, 48], and necessary for building the trust required to encourage behaviour change and non-directive goal-setting [48]. Knowledge of the local area was considered key for promoting collaborative working between primary care and community organisations [16, 33]. Furthermore, being proactive and tailoring the service to the needs of the local community were also regarded as essential to the success of social prescribing [38].

A reliance on the characteristics of individual link workers as drivers for success may pose a threat to the sustainability of social prescribing schemes given the role stresses described above which may threaten the retention of link workers in their role.

### The organisational level

The organisational realities into which link workers and social prescribing were implemented shaped experiences of the role. The extent to which primary care organisations bought into social prescribing, were able to provide support and space for link workers, as well as funding models and targets could influence the experience of link workers in social prescribing programmes.

#### Organisational buy-in to social prescribing

Across studies, the extent to which organisations within which link workers were based bought into or understood the service could have a considerable impact on how link workers experienced their role. “Social prescribing champions”, staff with an in-depth understanding of the remit of the role and who worked to embed social prescribing into practices, could facilitate link worker access to practice meetings, training sessions, and increase visibility of the social prescribing service in the practice [37, 44]. Strong collective leadership—where responsibility for social prescribing was shared between general practitioners (GPs, link workers and practice managers)—worked to integrate social prescribing into the practice and enabled link workers to be proactive and strategic with the community networking aspect of their role [31]. In cases where practices were not as engaged or did not fully understand what the service could offer, link workers had to take a more active role promoting the service, leaving less time to provide focused and holistic person-centred care [44, 48]. Co-locating link workers in primary care, providing space for them in practices, was noted to help referrers to understand and remember the link worker role [47], as well as making the service seem trustworthy and credible to patients/service users [33].

#### The pressure of targets

Organisational targets and service funding models that rewarded high referral volume and the completion of wellbeing assessments, could influence link workers’ approaches to patient care. These targets may cause a drift towards social prescribing approaches that prioritised the completion of assessment instruments at the expense of more holistic person-centred care [35, 44, 48]. Furthermore, the pressure to generate a high number of referrals could mean link workers accepted clients not necessarily ready to engage with social prescribing, or those with complex needs [48].

#### Support for link workers

To cope with high workloads, complex cases, and emotional burden, studies highlighted organisational support which could have a positive impact on link workers’ experiences of their role. The support available to link workers varied across the different contexts described in the studies. It was noted that link workers in larger organisations, with clear organisational structures, were more likely to be part of teams where workload could be shared and had better access to support and training [30]. Those working in teams with other link workers could draw on them for emotional support and reflection [45], and this could help promote a sense of success in the role [35]. Well-embedded workforce support and clinical supervision were key to being able to deal with the complex cases [37]. Where organisational support for link workers was not in place, some could find themselves isolated which could lead to feelings of anxiety and decreased capacity to cope [30, 37, 45].

### The system level

A key part of the link worker role is to facilitate service users’ social prescribing journeys from primary care to community groups and organisations. Social prescribing programmes depend on a “thriving” local community sector [32] that provides referral options for link workers to meet patient needs [38]. A number of the studies we reviewed drew attention to the fact that the wider health and social care system, as well as the voluntary and community sectors, were overstretched and underfunded [16, 32, 33, 35, 37, 38, 44, 48]. Several papers highlighted the impact of austerity, both on the availability of community and statutory services, and on the demand for them; describing it as a “perfect storm” [16] (p. e493) and a “threat to the future sustainability” [38] (p. 1541) of social prescribing.

#### A struggling wider health and social care system

Link workers sometimes felt like social prescribing was being used as a way to “boost up a crippled mental health service” [30] or as a “holding pen” [36], when other services that were over capacity, with long waiting lists, referred into social prescribing [37]. Fixsen et al. [33] talked about “changing demands”; they described how the pressure from closure of various local statutory and non-statutory services was compounded by the impact of the Covid-19 pandemic, and limited onward referral options for link workers. This meant that link workers needed to provide support for people with complex and/or severe mental health concerns, as well as support for those experiencing food poverty, unemployment, or needing benefits advice [37].

#### Local variation and structural poverty

Studies highlighted that the availability of services could vary across localities. Affluent areas were sometimes described as having an “abundance of community activities” [38], while those working in areas with high levels of socioeconomic disadvantage noted that this context limited their scope for onward referral [35]. Link workers made references to “structural poverty”, identifying the lack of investment in the local area [35]. Area level differences in service provision have implications for accessibility to services, in terms of requiring access to transport [35, 37, 38, 48] and being able to tailor support to patient needs [46], as well as with service users’ ability to engage with social prescribing due to complex life circumstances [44, 49].

#### Link workers as an additional resource

In the context of a system struggling from the effects of austerity, link workers were “welcomed as an extra resource” to tackle workload [16] (p. e491) or to deal with “the social” [40]. However, as described in earlier sections, link workers’ poorly defined role made them vulnerable to “filling gaps” [42].

The lack of onward referral options or other appropriate sources of support may mean that link workers end up taking a more involved approach to patient care than originally anticipated [38]. This may include taking on more risk than they have training for, due to having to deal with complex cases when there is no other support available [42]. There may also be a risk that patients develop “dependency” on their link worker in the absence of other support [47]. Having appropriate onward referral options was highlighted as key to helping to avoid patient dependency on the link worker [47], and to improving link workers’ ability to cope with high numbers of social prescribing referrals [48]. Providing longer-term and more intensive support may be counter to the original logic of social prescribing which seeks to signpost people away from primary care to appropriate community or statutory services [5].

### The service-user level

Social prescribing and the personalised care agenda [9] emphasise providing service users with “choice and control” and assume that they will be motivated and empowered to make changes that will benefit their health and wellbeing. However, across the reviewed studies, there was evidence to suggest that those experiencing precarious life circumstances may not be able to engage with social prescribing to the same extent as service users from more affluent backgrounds, highlighting that those in the most disadvantaged positions may be least likely to benefit. This calls into question social prescribing’s goal to help improve health equity. While more intensive and involved approaches to social prescribing may benefit disadvantaged groups, they require additional time, training, and support for link workers, and may not immediately deliver on social prescribing’s goal to reduce patient attendance in primary care.

#### Challenges to the dominant social prescribing discourses

Griffith et al. [35] observed how link workers’ narratives about their role were often ideological and conflicted, and drew on different and multiple social prescribing discourses. They discuss Mol’s work [51] and highlight the logics of “choice” and “care” dominant in social prescribing discourses among link workers, as well as in the policy logic of social prescribing. The “logic of choice” gives prominence to individual choice and control over services and support received. In the context of social prescribing, this means that service users have a range of options, and they are encouraged to take an active role in deciding which services and activities they would like to participate in. The onus is on individual service users to become “empowered”, through support provided by the link worker, to take control of their health, health behaviours, and personal situation. The “logic of care”, on the other hand, emphasises relational and holistic support, and focuses on building strong relationships with service users and tailoring support accordingly.

A dominant narrative among primary care staff in the reviewed papers was that poor health is caused by poor individual lifestyle choices, and that the role of social prescribing is to build the confidence of service users to empower them to make better decisions in relation to their health and wellbeing [40, 42], and to encourage “self-accountability” [36]. In some cases, it was suggested that service users needed to be able to self-refer to social prescribing as it signalled empowerment and autonomy. It was also argued that practitioner-led referral risked cultivating dependency [47] which could be considered a “threat to success” for social prescribing [36].

However, a number of studies drew attention to the various material and social circumstances that shaped the lives of service users and their ability to engage with social prescribing. The focus on empowerment and motivation may mean that service users from more affluent backgrounds are able to engage with the intervention and invest in their long-term health [44]. Social determinants of health shape the parameters within which individuals might be able to exercise choice and autonomy, as illustrated in this quotation from a participant in the study by Mackenzie et al. [40]: “life’s not great, they’ve got very little in the way of money now and they’re being squeezed and sanctioned ‘til they’re blue in the face. They haven’t got a job, they probably have to go and get a job, if they do … it’ll be like a zero-hour contract below the living wage … and so, to try and do that little bit extra about trying to live a more healthy lifestyle can just seem a bit pointless.” Furthermore, the local variation and lack of local investment and structural poverty described in the previous section may limit the “choice” of onward referrals for service users, further problematising the notions of choice and empowerment which are at the heart of some social prescribing discourses.

#### Temporal and material requirements for person-centred care

Despite the discourses emphasising personal empowerment, link workers do engage in approaches which can be interpreted as more aligned with the “logic of care”. Link workers often support service users with basic needs such as housing, homelessness, unemployment, and financial support [16, 38, 43, 44, 48]. They may also take on more intensive approaches by offering longer-term support [49], conducting home visits [41, 49], accompanying service users to activities or groups in the community [35, 44, 49], helping users from socioeconomically disadvantaged areas to navigate and access community services [48], and providing advocacy [35]. These more involved and longer-term approaches to social prescribing could be key in supporting individuals with multiple long-term conditions and experiences of socioeconomic deprivation [48].

However, these more involved approaches may require additional time and training for link workers, and may cause high levels of stress and emotional burden. Furthermore, as described in previous sections, the material and structural reality into which social prescribing has been placed may not be best suited to supporting such approaches to social prescribing. Such approaches may also not immediately deliver on social prescribing’s goal to reduce patient attendance in primary care as patients may require multiple visits over longer periods of time before feeling “empowered”.

### Line of argument

**Fig. 2:**
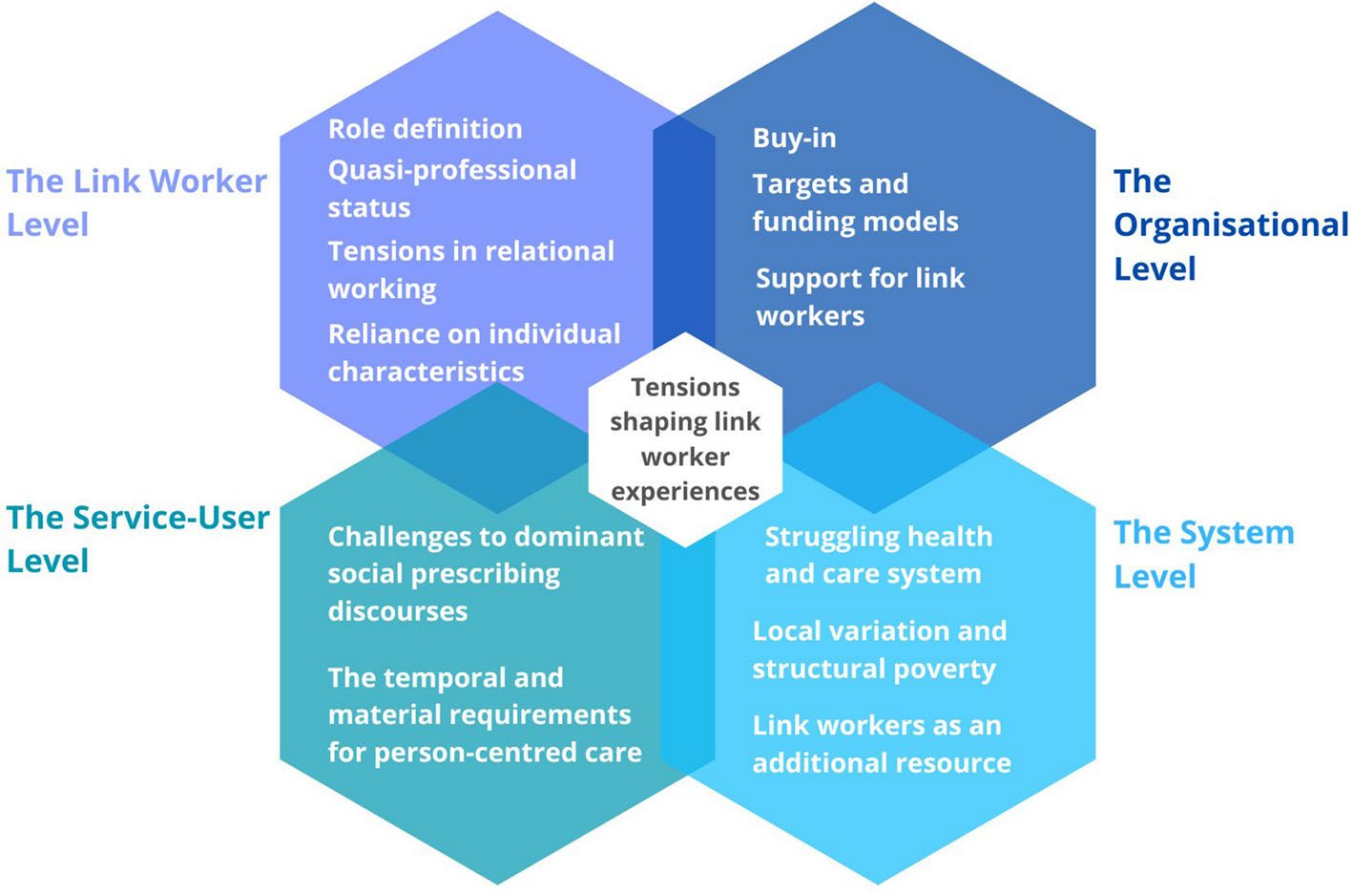
Visual summary of key components in the line of argument developed through this synthesis of qualitative literature. It depicts the different tensions at each of the levels at which social prescribing operates, which in turn shape link workers’ experiences of carrying out their role

Our analysis has highlighted inherent tensions present at each of the levels that social prescribing operates, which may arise from a mismatch between the policy logic around this initiative and the material and structural reality into which social prescribing is being implemented. These tensions may shape link workers’ experiences of their role and call into question the sustainability of social prescribing and the link worker role as currently implemented. A visual summary of the key components of our line of argument can be seen in Fig. 2.

At the link worker level, the way in which the role itself has been delineated and implemented may threaten the ability of social prescribing to deliver its intended outcomes. Link workers have been introduced into the health and care system as if they were a “professional” role, but without the formal training and registration that would afford them professional status. In its current form, the link worker role lacks strict definitions or boundaries. This flexibility in the role may allow link workers to tailor the way they work to fit with the population and area they serve, ultimately helping them to provide person-centred care. Conversely, it can cause role stress through workload overload and complexity, as well as confusion/ambivalence among other members of staff about what social prescribing is and what the link worker role can offer, in turn contributing to inappropriate referrals. In the context of an overstretched system, link workers may end up filling gaps and “holding” [52] service users they cannot move on into other more appropriate services. Enjoyment and fulfilment in the role appear to come from the fact that connecting with services users and supporting them on their journeys feels rewarding. The role stress that link workers may experience, coupled with low wages, may pose a threat to link worker retention. Given that much of the success of social prescribing seems to rely on individual link worker characteristics (such as being well-connected, knowledgeable about the local area, and having the skills needed to connect and build trusting relationships with service users), the role stress link workers experience, and associated implications for retention, may have implications for the success of social prescribing programmes.

The organisational contexts into which link workers and social prescribing are being implemented into can vary, influencing link workers’ experiences and the ways in which social prescribing is embedded into practice. Organisational buy-in, including support from leadership and champions who advocate for link workers and help to integrate them and the service into practices, appears to be essential. Furthermore, co-locating link workers in primary care settings may enhance their visibility and credibility. Organisational targets and funding models can shape a link worker’s role, sometimes leading to a focus on quantity of referrals rather than person-centred approaches. Support from organisations, including clear structures, teamwork, and access to training and supervision, are essential for link workers to manage their workload and address the emotional aspects of their roles effectively.

Social prescribing and the link worker role are about moving service users out of primary care, and into other sources of support which may be more appropriate [5]. The logic of the intervention relies on the presence and accessibility of onward referral options—community organisations and services, health and social care services, and statutory services. Yet, as discussed in several studies in this synthesis, decades of austerity have had an impact on both the availability of community and statutory services, as well as the demands for them through the impact it has had on individual livelihoods.

Social prescribing and the personalised care agenda [9] emphasise providing service users with “choice and control”, and assume that they will be motivated and empowered to make changes that will benefit their health and wellbeing. However, across the studies we reviewed, there was evidence to suggest that those experiencing precarious life circumstances may not be able to engage with the intervention to the same extent as service users from more affluent backgrounds. The pervasiveness of the discourses of choice and control may shift link workers away from more intensive person-centred approaches which may be more likely to benefit those experiencing difficult life circumstances and health inequities.

## Discussion

The aim of this meta-ethnographic synthesis was to develop a novel conceptual framework to understand the factors that shape link workers’ experiences and ways in which their role is being implemented. Our synthesis of 21 qualitative studies reveals that the lived reality of being a link worker appears to be shaped by tensions present at each of the levels at which social prescribing operates.

One of the dominant discourses, or policy logics, promotes social prescribing as a means of tackling health inequities through overcoming the social determinants of health and addressing failures of the health system to do so [6]. A number of the studies included in the synthesis discuss the impact that the long lasting effects of austerity have had both on the system-level infrastructure into which social prescribing and link workers have been introduced as well as on people’s livelihoods [16, 35, 40, 44, 49]. The “structural antecedents” [5] required for social prescribing to succeed have been shaped by political and economic forces which have seen cuts and disinvestment in voluntary and community as well as statutory organisations [53]. These cuts have disproportionately affected disadvantaged areas, where the need for services is typically greater [53, 54], and have worked to widen health inequalities [53]. The idea that social prescribing might mitigate the impact of austerity on healthcare by linking service users with community resources and encouraging collaborative working between organisations may therefore be questioned. The lived experience of link workers, as depicted in papers included in this review, highlights how the state of the wider health and care system both impacts their workloads and their role stress due to the fact that they may find themselves “holding” [52] service users who they cannot connect onwards due to relevant services or support either not being present or having limited capacity [35, 55]. Furthermore, facility-related pressures within GP surgeries [56] may mean that they are not able to provide link workers with physical space in practices [47]. The cuts in funding to public services may also have an impact on an organisation’s ability to adopt new healthcare innovations, such as social prescribing [57].

Other authors have highlighted the pervasiveness of neoliberal rhetoric present within social prescribing discourses [40, 58]. Neoliberalism is a political and economic ideology that emphasises market-based values such as individual choice, competition, economic liberalisation, privatisation, and profit maximisation. It is said to lead to policies that promote individual choice and responsibility, and commodification, which can have negative impacts on health and health equity [59]. Austerity measures, such as cutting social spending, align with neoliberal principles of minimising government involvement and reducing public services in order to promote economic growth. This can result in policies that prioritise budgetary constraints over social welfare [59]. The focus on individuality and empowerment places the onus on maintaining health on individuals and communities [35].

Social prescribing policies emphasise community-centred ways of working and asset-based community development [60]. These approaches focus on leveraging the strengths, skills and resources within a community, rather than focusing on its deficits, empowering communities to work collaboratively to address social issues in a way that fosters sustainable and positive change [61]. However, critics argue that these approaches can shift responsibility and resourcing away from the state and onto communities and may inadvertently contribute to inequality and justify cuts to social programmes [61].

In terms of factors that influence the ways in which link workers work with service users, the focus on individual choice and empowerment in social prescribing has been called “fantastical” [40], individualising health inequalities and targeting individual behaviours as the main solution, rather than addressing the fundamental causes of inequalities and ignoring the socio-political determinants of health [62]. As demonstrated in this review, the material and social circumstances of people’s lives can influence their ability to engage in social prescribing. In the context of austerity, where community services and infrastructure may be limited, “choice” and “control” can become misnomers. While those experiencing disadvantage may benefit from social prescribing, this may require a more involved and long-term approach from link workers that supersedes the 6–12 sessions over a 3-month period typically expected for social prescribing [63]. Evaluative work by the authors has identified that a lack of cohesion between what is expected in terms of patient turnover and what is needed to support people with challenging life circumstances can become a considerable source of tension for link workers, affecting their job satisfaction and retention [64].

Our review highlighted the role stress link workers experience through lack of a clear role definition and professional status. Role stress can occur when individuals face conflicting or incompatible expectations within a role they occupy, or when roles are unclear or poorly understood [65]. This strain arises from the challenges of balancing multiple and sometimes contradictory demands and expectations associated with the role. While reviewed papers suggested that role flexibility appeared to be a requirement for the delivery of person-centred and responsive care, the struggling infrastructure into which the link worker role has been established means it is at risk of being used to fill gaps. While a significant policy rationale for social prescribing is its potential to meet unmet needs and contribute to service development in local communities [5], gap-filling may expose link workers to more risk and complexity than they are prepared, trained, or compensated for, risking link worker retention [42]. In other contexts, it has been cautioned that “gap-filling” may undermine a profession’s attempt at establishing itself and being understood by others [66]. This may also make it difficult to feel valued or respected by colleagues due to the lack of appreciation of the role’s unique contribution to healthcare [66].

While the infrastructure that social prescribing and link workers have been implemented into may pose a number of challenges that threaten the sustainability of social prescribing, it is important to recognise that there are examples of social prescribing schemes that have thrived [67, 68] and that it can have positive outcomes in people’s lives [49]. Future research could explore the ways in which link workers navigate the challenges of structural and material realities they work in to deliver positive outcomes for service users.

### Strengths, limitations and reflexivity

This review is the first to synthesise published qualitative data of social prescribing link workers’ experiences of carrying out their social prescribing role in a way that develops a novel theoretical understanding. A key strength of this review lies in its meta-ethnographic approach. This led to the development of a “line of argument synthesis” which allowed us to move beyond the findings of individual studies to create a theory that is more than the sum of the parts included in the synthesis [69].

As with other qualitative syntheses, our review relied on the interpretations of the original study authors as well as their selection and presentation of study participant quotes [25]. We include participant quotes throughout our synthesis to help ensure it was grounded in participant experiences. Meta-ethnography was originally developed for synthesising meaning across ethnographies, which traditionally provide thick descriptions of the phenomena they are studying in order to contribute to a broader theoretical understanding. This thick description and focus on meaning, supports the development of third-order interpretations within a meta-ethnographic synthesis. As Atkins et al. [25] also found, qualitative research in health service research and public health is often more descriptive and applied and focused on providing evaluations and recommendations for policy and practice [70]. This meant that third-order interpretations may be more dependent on themes identified in studies than interpretations [25].

Social prescribing and the link worker role both have numerous definitions and operationalisations internationally [13]. Our search strategy attempted to account for differences in terminology internationally and tried to locate studies on interventions that met the operational definition of social prescribing, but which may not have referred to it as such. However, it is important to acknowledge that our review focused on link worker models of social prescribing which therefore may have limited included papers to those from United Kingdom (UK) settings where this model is now well-established [71].

The review team comprised a range of multidisciplinary expertise from social science, clinical practice, and health service, policy, and social prescribing research. This different expertise was helpful in developing the focus of the synthesis, and in the analysis process of translating and interpreting meaning across the different studies. The lead author (AT) has research interests in health inequities, the social determinants of health, and the social, economic, and political forces that drive them, which may have had an impact on how study findings were read and interpreted. However, emerging findings were discussed with a lay patient and public involvement group, as well as with a range of key social prescribing stakeholders in order to sense check interpretations as they were developing [72].

## Conclusions

This review has shown that link workers experience challenges in their role due to the structural realities (at a practice and broader level) within which the role has been developed and implemented, and these are also shaped by political and social forces. These challenges may call into question the sustainability of social prescribing due to the threat they pose to the retention of link workers in their roles, as well as the ability of local community infrastructure to support those in greatest need. The question of link worker retention is pertinent in the broader context of global healthcare workforce shortages where recruiting and retaining staff in key roles is already challenging [73].

Our review highlights a need for greater consideration of how the link worker role is defined, deployed, trained, and supported, which may affect recruitment and retention. It also highlights the need to ensure that the infrastructure it is being implemented into is sufficient to meet needs. Social prescribing models may require more intensive and longer-term modes of delivery to support those experiencing social and economic disadvantage. Further considerations ought to be given to ensuring that the community infrastructure is available and able to receive social prescribing referrals. A failure to acknowledge the wider reality of people and communities means that social prescribing risks being a neoliberal solution to problems neoliberalism has caused. Therefore, it may not be able to deliver its intended outcomes. Social prescribing should not be considered a substitute for broader social policy changes needed to address health inequities and ensure the equitable distribution of funding to areas and populations of greatest need.

### Supplementary Information

Additional file 1. Search strategy (PDF)

Additional file 2. Initial concept mapping (PDF)

Additional file 2. Critical Appraisal using the CASP tool (PDF)

## Data Availability

Datasets used in the review are available from the corresponding author upon request.

## Abbreviations

CASP: Critical Appraisal Skills Programme
CLP: Community links practitioner
GP: General practitioner
MND: Motor neuron disease
NHS: National Health Service
UK: United Kingdom
VCS: Voluntary and community sector
