# Supplementary material for: A meta-ethnography of the factors that shape link workers’ experiences of social prescribing
Source: BMC Med. 2024 Jul 4;22:280. doi: 10.1186/s12916-024-03478-w (PMC11225255; doi:10.1186/s12916-024-03478-w)
Supplement: Supplementary file 1 — Additional file 1. Search strategy (PDF) [file 12916_2024_3478_MOESM1_ESM.pdf]

Additional File 1: Detailed search stratehy for meta-ethnography

Medline:

Medline (Ovid MEDLINE® Epub Ahead of Print, In-Process & Other Non-Indexed Citations, Ovid MEDLINE® Daily and Ovid MEDLINE®) 1946 to present

1	social prescri*.ti,ab,kw.	247
2	(link? adj2 (worker? or practitioner? or staff or personnel or officer? or team?)).ti,ab,kw.	272
3	Patient Navigation/	870
4	((care or healthcare or community or outreach or patient?) adj5 navigator?).ti,ab,kw.	1041
5	(navigator? adj5 (program* or intervention* or service* or system?)).ti,ab,kw.	606
6	(((care or healthcare or community or outreach or patient?) adj2 navigat*) and (worker? or practitioner? or staff or personnel or team?)).ti,ab,kw.	586
7	((navigat* adj2 (program* or intervention* or service* or system?)) and (worker? or practitioner? or staff or personnel or team?)).ti,ab,kw.	556
8	community health workers/ or medical receptionists/	5980
9	(receptionist? or reception staff or reception personnel).ti,ab,kw.	544
10	(health* adj (assistant? or aide? or advisor? or adviser? or advocate? or co-ordinator? or coordinator? or connector? or officer? or facilitator? or liaison or broker? or coach* or promoter? or mobili?er?)).ti,ab,kw.	6539
11	(community adj (assistant? or aide? or advisor? or adviser? or advocate? or co-ordinator? or coordinator? or connector? or officer? or facilitator? or liaison or broker? or coach* or promoter? or mobili?er?)).ti,ab,kw.	999
12	8 or 9 or 10 or 11	13617
13	((navigat* or sign post* or signpost* or path* or guided or guiding or refer*) adj5 (service? or system? or care? or healthcare or patient? or community* or support)).ti,ab,kw.	331673
14	((navigat* or sign post* or signpost* or path* or guided or guiding or refer*) adj5 (group? or club? or selfhelp* or self-help or education or learning or exercise? or physical activity)).ti,ab,kw.	73969
15	((navigat* or sign post* or signpost* or path* or guided or guiding or refer*) adj5 (social support or social network? or social activit* or social service? or social resource? or community support or community network? or community activit* or community service? or community resource?)).ti,ab,kw.	1344
16	(active* adj3 (signpost* or sign post*)).ti,ab,kw.	7
17	(navigat* or sign post* or signpost*).ti.	14535
18	intersectoral collaboration/	2425
19	community-institutional relations/ or interinstitutional relations/	21262
20	interdisciplinary communication/	17881
21	(charities/ or faith-based organizations/ or organizations, nonprofit/ or voluntary health agencies/) and (link* or liais* or collaborat* or partner* or engage* or leverag* or broker* or coordinat* or co-ordinat*).ti,ab,kw.	1076

22	((multidisciplin* or interdisciplin* or multi* disciplin* or inter disciplin* or multisector* or intersector* or multi* sector or inter sector* or multiorgani?ation* or interorgani?ation* or multi* organi?ation* or inter organi?ation*) and (link* or liais* or collaborat* or partner* or engage* or leverag* or broker* or coordinat* or co-ordinat*)).ti,ab,kw.	32776
23	(((voluntary or volunteer* or charit* or faith* or church* or community or third or nongovernment* or non-government* or nonprofit* or non-profit* or "not for profit*") adj2 (sector? or agenc* or organi?ation?)) and (link* or liais* or collaborat* or partner* or engage* or leverag* or broker* or coordinat* or co-ordinat*)).ti,ab,kw.	8269
24	Social Determinants of Health/	4558
25	Health Equity/	2044
26	(social determinant? or social need? or psychosocial need?).ti,ab,kw.	13162
27	((health or social or psychosocial or economic) adj2 (equit* or inequit* or equal* or inequal* or depriv*)).ti,ab,kw.	25642
28	((social* adj2 (interact* or isolat*)) or loneliness or lonely).ti,ab,kw.	42696
29	13 or 14 or 15 or 16 or 17 or 18 or 19 or 20 or 21 or 22 or 23 or 24 or 25 or 26 or 27 or 28	553565
30	12 and 29	1800
31	3 or 4 or 5 or 6 or 7 or 30	4181
32	general practice/ or family practice/	76359
33	general practitioners/ or physicians, family/ or physicians, primary care/	29155
34	Primary Health Care/	83443
35	Office Visits/	7218
36	(ambulatory adj3 (care or setting? or facilit* or ward? or department? or service?)).ti,ab,kw.	18694
37	((general or family) adj2 (practi* or physician? or doctor?)).ti,ab,kw.	123954
38	(primary care or primary health care or primary healthcare).ti,ab,kw.	153225
39	(clinic? or visit?).ti,ab,kw.	551099
40	((health* or medical) adj2 (center? or centre?)).ti,ab,kw.	137168
41	Community Health Services/	32425
42	community.ti.	151984
43	(community adj3 (service? or care or health*)).ti,ab,kw.	82400
44	32 or 33 or 34 or 35 or 36 or 37 or 38 or 39 or 40 or 41 or 42 or 43	1107504
45	31 and 44	2234
46	1 or 2 or 45	2708
47	exp qualitative research/	66831
48	focus groups/ or interviews as topic/	93850
49	grounded theory/	2126
50	qualitative.ti. or (qualitative adj2 (stud* or research or analysis)).ti,ab,kw.	114984

Additional File 1: Detailed search stratehy for meta-ethnography

51	(interview* or focus group* or mixed method* or multiple method* or multimethod* or multi-method*).ti,ab,kw.	436089
52	(grounded theory or ethnograph* or ipa or phenomenolog* or thematic analysis or narrative analysis).ti,ab,kw.	83844
53	47 or 48 or 49 or 50 or 51 or 52	542917
54	46 and 53	946
55	afghanistan/ or exp africa/ or albania/ or andorra/ or antarctic regions/ or argentina/ or exp asia, central/ or exp asia, northern/ or exp asia, southeastern/ or exp atlantic islands/ or bahrain/ or bangladesh/ or bhutan/ or bolivia/ or borneo/ or "bosnia and herzegovina"/ or brazil/ or bulgaria/ or exp central america/ or exp china/ or colombia/ or "commonwealth of independent states"/ or croatia/ or "democratic people's republic of korea"/ or ecuador/ or gibraltar/ or guyana/ or exp india/ or indonesia/ or iran/ or iraq/ or jordan/ or kosovo/ or kuwait/ or lebanon/ or liechtenstein/ or macau/ or "macedonia (republic)"/ or exp melanesia/ or moldova/ or monaco/ or mongolia/ or montenegro/ or nepal/ or netherlands antilles/ or new guinea/ or oman/ or pakistan/ or paraguay/ or peru/ or philippines/ or qatar/ or "republic of belarus"/ or romania/ or exp russia/ or saudi arabia/ or serbia/ or sri lanka/ or suriname/ or syria/ or taiwan/ or exp transcaucasia/ or ukraine/ or uruguay/ or united arab emirates/ or exp ussr/ or venezuela/ or yemen/	1198703
56	organisation for economic co-operation and development/	367
57	australasia/ or exp australia/ or austria/ or exp baltic states/ or belgium/ or exp canada/ or chile/ or czech republic/ or europe/ or exp france/ or exp germany/ or greece/ or hungary/ or ireland/ or israel/ or exp italy/ or exp japan/ or korea/ or luxembourg/ or mexico/ or netherlands/ or new zealand/ or north america/ or poland/ or portugal/ or exp "republic of korea"/ or exp "scandinavian and nordic countries"/ or slovakia/ or slovenia/ or spain/ or switzerland/ or turkey/ or exp united kingdom/ or exp united states/	3301585
58	european union/	16805
59	developed countries/	20884
60	56 or 57 or 58 or 59	3316510
61	55 not 60	1111220
62	54 not 61	722

Additional File 1: Detailed search stratehy for meta-ethnography

Embase:

	Embase 1974 to present	
1	social prescri*.ti,ab,kw.	284
2	(link? adj2 (worker? or practitioner? or staff or personnel or officer? or team?)).ti,ab,kw.	363
3	((care or healthcare or community or outreach or patient?) adj5 navigator?).ti,ab,kw.	2241
4	(navigator? adj5 (program* or intervention* or service* or system?)).ti,ab,kw.	1108
5	(((care or healthcare or community or outreach or patient?) adj2 navigat*) and (worker? or practitioner? or staff or personnel or team?)).ti,ab,kw.	1333
6	((navigat* adj2 (program* or intervention* or service* or system?)) and (worker? or practitioner? or staff or personnel or team?)).ti,ab,kw.	1026
7	care coordinator/ or health auxiliary/ or lay health worker/ or medical receptionist/	8239
8	(receptionist? or reception staff or reception personnel).ti,ab,kw.	768
9	(health* adj (assistant? or aide? or advisor? or adviser? or advocate? or co-ordinator? or coordinator? or connector? or officer? or facilitator? or liaison or broker? or coach* or promoter? or mobili?er?)).ti,ab,kw.	7990
10	(community adj (assistant? or aide? or advisor? or adviser? or advocate? or co-ordinator? or coordinator? or connector? or officer? or facilitator? or liaison or broker? or coach* or promoter? or mobili?er?)).ti,ab,kw.	1391
11	7 or 8 or 9 or 10	17918
12	((navigat* or sign post* or signpost* or path* or guided or guiding or refer*) adj5 (service? or system? or care? or healthcare or patient? or community* or support)).ti,ab,kw.	535424
13	((navigat* or sign post* or signpost* or path* or guided or guiding or refer*) adj5 (group? or club? or selfhelp* or self-help or education or learning or exercise? or physical activity)).ti,ab,kw.	108517
14	((navigat* or sign post* or signpost* or path* or guided or guiding or refer*) adj5 (social support or social network? or social activit* or social service? or social resource? or community support or community network? or community activit* or community service? or community resource?)).ti,ab,kw.	1878
15	(active* adj3 (signpost* or sign post*)).ti,ab,kw.	10
16	(navigat* or sign post* or signpost*).ti.	18328
17	intersectoral collaboration/	2773
18	interdisciplinary communication/	12787

19	(social welfare/ or faith-based organization/ or non-governmental organization/ or sports organization/) and (link* or liais* or collaborat* or partner* or engage* or leverag* or broker* or coordinat* or co-ordinat*).ti,ab,kw.	3616
20	((multidisciplin* or interdisciplin* or multi* disciplin* or inter disciplin* or multisector* or intersector* or multi* sector or inter sector* or multiorgani?ation* or interorgani?ation* or multi* organi?ation* or inter organi?ation*) and (link* or liais* or collaborat* or partner* or engage* or leverag* or broker* or coordinat* or co-ordinat*)).ti,ab,kw.	53092
21	(((voluntary or volunteer* or charit* or faith* or church* or community or third or nongovernment* or non-government* or nonprofit* or non-profit* or "not for profit*") adj2 (sector? or agenc* or organi?ation?)) and (link* or liais* or collaborat* or partner* or engage* or leverag* or broker* or coordinat* or co-ordinat*)).ti,ab,kw.	10527
22	social determinants of health/	11081
23	Health Equity/	4960
24	(social determinant? or social need? or psychosocial need?).ti,ab,kw.	18356
25	((health or social or psychosocial or economic) adj2 (equit* or inequit* or equal* or inequal* or depriv*)).ti,ab,kw.	31704
26	((social* adj2 (interact* or isolat*)) or loneliness or lonely).ti,ab,kw.	53981
27	12 or 13 or 14 or 15 or 16 or 17 or 18 or 19 or 20 or 21 or 22 or 23 or 24 or 25 or 26	804752
28	11 and 27	2859
29	3 or 4 or 5 or 6 or 28	6566
30	general practice/	79758
31	general practitioner/	105374
32	*primary medical care/ or *primary health care/	64986
33	(ambulatory adj3 (care or setting? or facilit* or ward? or department? or service?)).ti,ab,kw.	27112
34	((general or family) adj2 (practi* or physician? or doctor?)).ti,ab,kw.	156933
35	(primary care or primary health care or primary healthcare).ti,ab,kw.	202130
36	(clinic? or visit?).ti,ab,kw.	879283
37	((health* or medical) adj2 (center? or centre?)).ti,ab,kw.	203651
38	community care/	56315
39	community.ti.	177187
40	(community adj3 (service? or care or health*)).ti,ab,kw.	105307
41	30 or 31 or 32 or 33 or 34 or 35 or 36 or 37 or 38 or 39 or 40	1594306
42	29 and 41	3867
43	1 or 2 or 42	4463
44	exp qualitative research/	91441
45	multimethod study/ or open ended questionnaire/ or exp interview/	315010

46	grounded theory/	8446
47	qualitative.ti. or (qualitative adj2 (stud* or research or analysis)).ti,ab,kw.	145359
48	(interview* or focus group* or mixed method* or multiple method* or multimethod* or multi-method*).ti,ab,kw.	551514
49	(grounded theory or ethnograph* or ipa or phenomenolog* or thematic analysis or narrative analysis).ti,ab,kw.	101486
50	44 or 45 or 46 or 47 or 48 or 49	722635
51	43 and 50	1411
52	afghanistan/ or exp africa/ or argentina/ or bahrain/ or bangladesh/ or bhutan/ or bolivia/ or borneo/ or exp brazil/ or brunei darussalam/ or exp china/ or colombia/ or ecuador/ or equatorial guinea/ or guinea/ or guinea-bissau/ or guyana/ or exp india/ or exp indonesia/ or iran/ or exp iraq/ or jordan/ or kazakhstan/ or kuwait/ or kyrgyzstan/ or laos/ or lebanon/ or exp malaysia/ or mongolia/ or myanmar/ or nepal/ or north korea/ or oman/ or exp pakistan/ or papua new guinea/ or paraguay/ or peru/ or qatar/ or philippines/ or exp russian federation/ or saudi arabia/ or singapore/ or sri lanka/ or suriname/ or syrian arab republic/ or taiwan/ or tajikistan/ or thailand/ or timor-leste/ or turkmenistan/ or exp united arab emirates/ or uruguay/ or exp uzbekistan/ or venezuela/ or viet nam/ or yemen/	1366252
53	exp "organisation for economic co-operation and development"/	1794
54	exp "australia and new zealand"/ or austria/ or exp "baltic states"/ or exp belgium/ or chile/ or "czech republic"/ or denmark/ or europe/ or exp finland/ or exp france/ or exp germany/ or greece/ or hungary/ or iceland/ or ireland/ or israel/ or exp italy/ or japan/ or korea/ or luxembourg/ or netherlands/ or exp "north america"/ or exp norway/ or exp portugal/ or scandinavia/ or slovakia/ or slovenia/ or "south korea"/ or exp spain/ or sweden/ or switzerland/ or exp "turkey (republic)"/ or exp united kingdom/ or western europe/	3402016
55	european union/	28619
56	developed country/	34184
57	53 or 54 or 55 or 56	3432758
58	52 not 57	1249567
59	51 not 58	1111

Additional File 1: Detailed search stratehy for meta-ethnography

PsycINFO

	PsycINFO 1806 to present	
1	social prescri*.ti,ab.	97
2	(link? adj2 (worker? or practitioner? or staff or personnel or officer? or team?)).ti,ab.	226
3	((care or healthcare or community or outreach or patient?) adj5 navigator?).ti,ab.	303
4	(navigator? adj5 (program* or intervention* or service* or system?)).ti,ab.	176
5	(((care or healthcare or community or outreach or patient?) adj2 navigat*) and (worker? or practitioner? or staff or personnel or team?)).ti,ab.	226
6	((navigat* adj2 (program* or intervention* or service* or system?)) and (worker? or practitioner? or staff or personnel or team?)).ti,ab.	225
7	exp clerical personnel/	713
8	(receptionist? or reception staff or reception personnel).ti,ab.	165
9	(health* adj (assistant? or aide? or advisor? or adviser? or advocate? or co-ordinator? or coordinator? or connector? or officer? or facilitator? or liaison or broker? or coach* or promoter? or mobili?er?)).ti,ab.	1870
10	(community adj (assistant? or aide? or advisor? or adviser? or advocate? or co-ordinator? or coordinator? or connector? or officer? or facilitator? or liaison or broker? or coach* or promoter? or mobili?er?)).ti,ab.	585
11	7 or 8 or 9 or 10	3317
12	((navigat* or sign post* or signpost* or path* or guided or guiding or refer*) adj5 (service? or system? or care? or healthcare or patient? or community* or support)).ti,ab.	48596
13	((navigat* or sign post* or signpost* or path* or guided or guiding or refer*) adj5 (group? or club? or selfhelp* or self-help or education or learning or exercise? or physical activity)).ti,ab.	25303
14	((navigat* or sign post* or signpost* or path* or guided or guiding or refer*) adj5 (social support or social network? or social activit* or social service? or social resource? or community support or community network? or community activit* or community service? or community resource?)).ti,ab.	1252
15	(active* adj3 (signpost* or sign post*)).ti,ab.	2
16	(navigat* or sign post* or signpost*).ti.	5095
17	(ngos/ or nonprofit organizations/ or exp religious organizations/) and (link* or liais* or collaborat* or partner* or engage* or leverag* or broker* or coordinat* or co-ordinat*).ti,ab.	1596

18	((multidisciplin* or interdisciplin* or multi* disciplin* or inter disciplin* or multisector* or intersector* or multi* sector or inter sector* or multiorgani?ation* or interorgani?ation* or multi* organi?ation* or inter organi?ation*) and (link* or liais* or collaborat* or partner* or engage* or leverag* or broker* or coordinat* or co-ordinat*)).ti,ab.	14424
19	(((voluntary or volunteer* or charit* or faith* or church* or community or third or nongovernment* or non-government* or nonprofit* or non-profit* or "not for profit*") adj2 (sector? or agenc* or organi?ation?)) and (link* or liais* or collaborat* or partner* or engage* or leverag* or broker* or coordinat* or co-ordinat*)).ti,ab.	6328
20	health disparities/	9009
21	(social determinant? or social need? or psychosocial need?).ti,ab.	6634
22	((health or social or psychosocial or economic) adj2 (equit* or inequit* or equal* or inequal* or depriv*)).ti,ab.	12999
23	((social* adj2 (interact* or isolat*)) or loneliness or lonely).ti,ab.	56425
24	12 or 13 or 14 or 15 or 16 or 17 or 18 or 19 or 20 or 21 or 22 or 23	174481
25	11 and 24	377
26	3 or 4 or 5 or 6 or 25	1027
27	family medicine/	1274
28	general practitioners/ or family physicians/	7579
29	(ambulatory adj3 (care or setting? or facilit* or ward? or department? or service?)).ti,ab.	2249
30	((general or family) adj2 (practi* or physician? or doctor?)).ti,ab.	24170
31	(primary care or primary health care or primary healthcare).ti,ab.	35484
32	(clinic? or visit?).ti,ab.	107563
33	((health* or medical) adj2 (center? or centre?)).ti,ab.	22331
34	community health/ or community services/	20090
35	community.ti.	63600
36	(community adj3 (service? or care or health*)).ti,ab.	40826
37	27 or 28 or 29 or 30 or 31 or 32 or 33 or 34 or 35 or 36	255285
38	26 and 37	469
39	1 or 2 or 38	781
40	exp qualitative methods/ or mixed methods research/	18189
41	interviews/ or focus group interview/ or semi-structured interview/	12199
42	qualitative.ti. or (qualitative adj2 (stud* or research or analysis)).ti,ab.	99715
43	(interview* or focus group* or mixed method* or multiple method* or multimethod* or multi-method*).ti,ab.	387828
44	(grounded theory or ethnograph* or ipa or phenomenolog* or thematic analysis or narrative analysis).ti,ab.	109288
45	40 or 41 or 42 or 43 or 44	477990
46	39 and 45	286

47	developing countries/ not developed countries/	5412
48	46 not 47	286

CINAHL

#	Query	Results
S33	S26 NOT S32	1.046
S32	S27 NOT S31	266.625
S31	S28 OR S29 OR S30	1.629.324
S30	(MH "European Union") OR (MH "Developed Countries")	9.787
S29	(MH "Australia+") OR (MH "New Zealand") OR (MH "North America") OR (MH "Canada+") OR (MH "Mexico") OR (MH "United States+") OR (MH "Austria") OR (MH "Belgium") OR (MH "Germany+") OR (MH "France") OR (MH "Greece") OR (MH "Ireland") OR (MH "Italy") OR (MH "Luxembourg") OR (MH "Netherlands") OR (MH "Portugal") OR (MH "Scandinavia+") OR (MH "Spain") OR (MH "Switzerland") OR (MH "United Kingdom+") OR (MH "Baltic States+") OR (MH "Czech Republic") OR (MH "Hungary") OR (MH "Poland") OR (MH "Japan") OR (MH "South Korea") OR (MH "Chile") OR (MH "Israel") OR (MH "Turkey")	1.622.390
S28	(MH "Organisation for Economic Co-Operation and Development")	195
S27	(MH "Africa") OR (MH "Antarctic Regions") OR (MH "Asia, Central+") OR (MH "Asia, Southeastern+") OR (MH "Bangladesh") OR (MH "Bhutan") OR (MH "India") OR (MH "Afghanistan") OR (MH "Bahrain") OR (MH "Iran") OR (MH "Jordan") OR (MH "Lebanon") OR (MH "Oman") OR (MH "Saudi Arabia") OR (MH "Syria") OR (MH "United Arab Emirates") OR (MH "Yemen") OR (MH "Nepal") OR (MH "Sri Lanka") OR (MH "Taiwan") OR (MH "North Korea") OR (MH "Mongolia") OR (MH "Macao") OR (MH "Andorra") OR (MH "China+") OR (MH "Albania") OR (MH "Bosnia-Herzegovina") OR (MH "Bulgaria") OR (MH "Byelarus") OR (MH "Croatia") OR (MH "Macedonia (Republic)") OR (MH "Moldova") OR (MH "Russia") OR (MH "Serbia") OR (MH "Ukraine") OR (MH "Yugoslavia") OR (MH "Central America+") OR (MH "Latin America") OR (MH "South America") OR (MH "Bolivia") OR (MH "Brazil") OR (MH "Ecuador") OR (MH "Guyana") OR (MH "Paraguay") OR (MH "Peru") OR (MH "Suriname") OR (MH "Uruguay") OR (MH "Venezuela") OR (MH "West Indies+") OR (MH "Atlantic Islands+") OR (MH "Low and Middle Income Countries") OR (MH "Melanesia+") OR (MH "Micronesia+") OR (MH "Polynesia+")	296.208
S26	S22 AND S25	1.159
S25	S23 OR S24	436.365

Additional File 1: Detailed search stratehy for meta-ethnography

S24	TI qualitative OR AB ((qualitative N2 (stud* or research or analysis))) OR TI ((interview* or "focus group*" or "mixed method*" or "multiple method*" or multimethod* or "multi-method*")) OR AB ((interview* or "focus group*" or "mixed method*" or "multiple method*" or multimethod* or "multi-method*")) OR TI (("grounded theory" or ethnograph* or ipa or phenomenolog* or "thematic analysis" or "narrative analysis")) OR AB (("grounded theory" or ethnograph* or ipa or phenomenolog* or "thematic analysis" or "narrative analysis"))	313.725
S23	(MH "Qualitative Studies+") OR (MH "Interviews+") OR (MH "Focus Groups")	312.069
S22	S1 OR S21	3.484
S21	S15 AND S20	2.496
S20	S16 OR S17 OR S18 OR S19	511.753
S19	TI community OR AB ((community N3 (service# or care or health*)))	131.511
S18	(MH "Community Health Services") OR (MH "Community Service")	24.278
S17	TI ((ambulatory N3 (care or setting# or facilit* or ward# or department# or service#))) OR AB ((ambulatory N3 (care or setting# or facilit* or ward# or department# or service#))) OR TI (((general or family) N2 (practi* or physician# or doctor#))) OR AB (((general or family) N2 (practi* or physician# or doctor#))) OR TI (("primary care" or "primary health care" or "primary healthcare")) OR AB (("primary care" or "primary health care" or "primary healthcare")) OR TI (clinic# or visit#) OR AB (clinic# or visit#) OR TI (((health* or medical) N2 (center# or centre#))) OR AB (((health* or medical) N2 (center# or centre#)))	353.798
S16	(MH "Family Practice") OR (MH "Physicians, Family") OR (MH "Primary Health Care") OR (MH "Office Visits")	109.462
S15	S2 OR S3 OR S14	6.149
S14	S6 AND S13	3.345
S13	S7 OR S8 OR S9 OR S10 OR S11 OR S12	231.079
S12	TI (("social determinant#" or "social need#" or "psychosocial need#")) OR AB (("social determinant#" or "social need#" or "psychosocial need#")) OR TI (((health or social or psychosocial or economic) N2 (equit* or inequit* or equal* or inequal* or depriv*))) OR AB (((health or social or psychosocial or economic) N2 (equit* or inequit* or equal* or inequal* or depriv*))) OR TI (((social* N2 (interact* or isolat*)) or loneliness or lonely)) OR AB (((social* N2 (interact* or isolat*)) or loneliness or lonely))	44.097
S11	(MH "Social Determinants of Health")	7.422

Additional File 1: Detailed search stratehy for meta-ethnography

S10	TI (((multidisciplin* or interdisciplin* or "multi* disciplin*" or "inter* disciplin*" or multisector* or intersector* or "multi* sector*" or "inter* sector*" or multiorgani#ation* or interorgani#ation* or "multi* organi#ation*" or "inter* organi#ation*") and (link* or liais* or collaborat* or partner* or engage* or leverag* or broker* or coordinat* or co-ordinat*))) OR AB (((multidisciplin* or interdisciplin* or "multi* disciplin*" or "inter* disciplin*" or multisector* or intersector* or "multi* sector*" or "inter* sector*" or multiorgani#ation* or interorgani#ation* or "multi* organi#ation*" or "inter* organi#ation*") and (link* or liais* or collaborat* or partner* or engage* or leverag* or broker* or coordinat* or co-ordinat*))) OR TI ((((voluntary or volunteer* or charit* or faith* or church* or community or third or nongovernment* or non-government* or nonprofit* or non-profit* or "not for profit") N2 (sector# or agenc* or organi#ation#)) and (link* or liais* or collaborat* or partner* or engage* or leverag* or broker* or coordinat* or co-ordinat*))) OR AB ((((voluntary or volunteer* or charit* or faith* or church* or community or third or nongovernment* or non-government* or nonprofit* or non-profit* or "not for profit") N2 (sector# or agenc* or organi#ation#)) and (link* or liais* or collaborat* or partner* or engage* or leverag* or broker* or coordinat* or co-ordinat*)))	21.592
S9	(MH "Charities") OR (MH "Organizations, Nonprofit") OR (MH "Voluntary Health Agencies+") OR (MH "Sports Organizations") OR (MH "Support Groups") AND ((TI (link* or liais* or collaborat* or partner* or engage* or leverag* or broker* or coordinat* or co-ordinat*)) OR (AB(link* or liais* or collaborat* or partner* or engage* or leverag* or broker* or coordinat* or co-ordinat*)))	23.665
S8	(MH "Community-Institutional Relations") OR (MH "Interinstitutional Relations")	14.028
S7	TI (((navigat* or sign post* or signpost* or path* or guided or guiding or refer*) N5 (service# or system# or care# or healthcare or patient# or community* or support))) OR AB (((navigat* or sign post* or signpost* or path* or guided or guiding or refer*) N5 (service# or system# or care# or healthcare or patient# or community* or support))) OR TI (((navigat* or sign post* or signpost* or path* or guided or guiding or refer*) N5 (group# or club# or selfhelp* or self-help or education or learning or exercise# or "physical activity"))) OR AB (((navigat* or sign post* or signpost* or path* or guided or guiding or refer*) N5 (group# or club# or selfhelp* or self-help or education or learning or exercise# or "physical activity"))) OR TI (((navigat* or sign post* or signpost* or path* or guided or guiding or refer*) N5 ("social support" or "social network*" or "social activit*" or "social service*" or "social resource*" or "community support" or "community network*" or "community activit*" or "community service*" or "community resource*"))) OR AB (((navigat* or sign post* or signpost* or path* or guided or guiding or refer*) N5 ("social support" or "social network*" or "social activit*" or "social service*" or "social resource*" or "community support" or "community network*" or "community activit*" or "community service*" or "community resource*"))) OR TI ((navigat* or "sign post*" or signpost*)) OR AB ((active* N3 (signpost* or "sign post*")))	130.975

Additional File 1: Detailed search stratehy for meta-ethnography

S6	S4 OR S5	27.960
S5	TI (("receptionist#" or "reception staff" or "reception personnel")) OR AB (("receptionist#" or "reception staff" or "reception personnel")) OR TI ((health* N1 (assistant# or aide# or advisor# or adviser# or advocate# or co-ordinator# or coordinator# or connector# or officer# or facilitator# or liaison or broker# or coach* or promoter#))) OR AB ((health* N1 (assistant# or aide# or advisor# or adviser# or advocate# or co-ordinator# or coordinator# or connector# or officer# or facilitator# or liaison or broker# or coach* or promoter# or mobili#er#))) OR TI ((community N1 (assistant# or aide# or advisor# or adviser# or advocate# or co-ordinator# or coordinator# or connector# or officer# or facilitator# or liaison or broker# or coach* or promoter#))) OR AB ((community N1 (assistant# or aide# or advisor# or adviser# or advocate# or co-ordinator# or coordinator# or connector# or officer# or facilitator# or liaison or broker# or coach* or promoter# or mobili#er#)))	9.596
S4	(MH "Community Health Workers") OR (MH "Clerical Personnel") OR (MH "Volunteer Workers")	18.734
S3	TI (((care or healthcare or community or outreach or patient#) N5 navigator#)) OR AB (((care or healthcare or community or outreach or patient#) N5 navigator#)) OR TI ((navigator# N5 (program* or intervention* or service* or system#))) OR AB ((navigator# N5 (program* or intervention* or service* or system#))) OR TI ((((care or healthcare or community or outreach or patient#) N2 navigat*) and (worker# or practitioner# or staff or personnel or team#))) OR AB ((((care or healthcare or community or outreach or patient#) N2 navigat*) and (worker# or practitioner# or staff or personnel or team#))) OR TI (((navigat* N2 (program* or intervention* or service* or system#)) and (worker# or practitioner# or staff or personnel or team#))) OR AB (((navigat* N2 (program* or intervention* or service* or system#)) and (worker# or practitioner# or staff or personnel or team#)))	1.895
S2	(MH "Patient Navigation")	1.541
S1	TX social prescri* OR TX ((link# N2 (worker# or practitioner# or staff or personnel or officer# or team#)))	1.007

Additional File 1: Detailed search stratehy for meta-ethnography

SociologyCollection

Set	**Search**	**Results**
S25	S18 OR S19 OR S2O OR S21 OR S22 OR S23 OR S24	**756**
**S24**	S15 AND S17	314
**S23**	S14 AND S17	285
**S22**	S13 AND S17	31
**S21**	S12 AND S17	37
**S20**	S11 AND S17	46
**S19**	S10 AND S17	33
**S18**	S2 AND S17	194
**S17**	TI(qualitative) or AB,TI(qualitative NEAR/2 (stud* or research or analysis)) OR AB,TI(interview* or ("focus group" OR "focus groups") or ("mixed method" OR "mixed methods") or ("multiple methods") or multimethod* or multi-method*) OR AB,TI("grounded theory" or ethnograph* or ipa or phenomenolog* or "thematic analysis" or "narrative analysis")	401833
**S15**	S8 AND S9	948
**S14**	S7 AND S9	911
**S13**	S6 AND S9	101
**S12**	S5 AND S9	76
**S11**	S4 AND S9	107
**S10**	S3 AND S9	123
**S9**	AB,TI(ambulatory NEAR/3 (care or setting?? or facilit* or ward?? or department?? or service??)) OR AB,TI((general or family) NEAR/2 (practi* or physician?? or doctor??)) OR AB,TI("primary care" or "primary health care" or "primary healthcare") OR AB,TI("clinic" OR "clinics" OR "visit" OR "visits") OR AB,TI((health* or medical) NEAR/2 (center?? or centre??)) OR TI(community) OR AB,TI(community NEAR/3 (service?? or care or health*))	223949
**S8**	AB,TI(community NEAR/1 (assistant?? or aide?? or advisor?? or adviser?? or advocate?? or co-ordinator?? or coordinator?? or connector?? or officer?? or facilitator?? or liaison or broker?? or coach* or promoter?? or mobili?er??))	1559
**S7**	AB,TI(health* NEAR/1 (assistant?? or aide?? or advisor?? or adviser?? or advocate?? or co-ordinator?? or coordinator?? or connector?? or officer?? or facilitator?? or liaison or broker?? or coach* or promoter?? or mobili?er??))	3673
**S6**	AB,TI(receptionist?? or "reception staff" or "reception personnel")	186

**S5**	AB,TI(navigat* NEAR/2 (program* or intervention* or service* or system??)) and AB,TI(worker?? or practitioner?? or staff or personnel or team??)	232
**S4**	AB,TI((care or healthcare or community or outreach or patient??) NEAR/2 navigat*) AND AB,TI(worker?? or practitioner?? or staff or personnel or team??)	219
**S3**	AB,TI((care or healthcare or community or outreach or patient??) NEAR/5 navigator??) OR AB,TI(navigator?? NEAR/5 (program* or intervention* or service* or system??))	293
**S2**	AB,TI("social prescri*") OR AB,TI(link?? NEAR/2 (worker?? or practitioner?? or staff or personnel or officer?? or team??))	734

SocSci

1	TS=("social prescri*" OR (link$ NEAR/2 (worker$ or practitioner$ or staff or personnel or officer$ or team$)))	1056
2	TS=((care or healthcare or community or outreach or patient$) NEAR/5 navigator$) OR TS=(navigator$ NEAR/5 (program* or intervention* or service* or system$)) OR TS=(((care or healthcare or community or outreach or patient$) NEAR/2 navigat*) and (worker$ or practitioner$ or staff or personnel or team?)) OR TS=((navigat* NEAR/2 (program* or intervention* or service* or system$)) and (worker$ or practitioner$ or staff or personnel or team$))	1290
3	TS=(receptionist$ or "reception staff" or "reception personnel") OR TS=(health* NEAR/1 (assistant$ or aide$ or advisor$ or adviser$ or advocate$ or co-ordinator$ or coordinator$ or connector$ or officer$ or facilitator$ or liaison or broker$ or coach* or promoter$ or mobili?er$)) OR TS=(community NEAR/1 (assistant$ or aide$ or advisor$ or adviser$ or advocate$ or co-ordinator$ or coordinator$ or connector$ or officer$ or facilitator$ or liaison or broker$ or coach* or promoter$ or mobili?er$))	6984
4	TS=((navigat* or "sign post*" or signpost* or path* or guided or guiding or refer*) NEAR/5 (service$ or system$ or care$ or healthcare or patient$ or community* or support)) OR TS=((navigat* or "sign post*" or signpost* or path* or guided or guiding or refer*) NEAR/5 (group$ or club$ or selfhelp* or self-help or education or learning or exercise$ or "physical activity")) OR TS=((navigat* or "sign post*" or signpost* or path* or guided or guiding or refer*) NEAR/5 ("social support" or "social network$" or "social activit*" or "social service$" or "social resource$" or "community support" or "community network$" or "community activit*" or "community service$" or "community resource$")) OR TS=(active* NEAR/3 (signpost* or "sign post*")) OR TI=(navigat* or "sign post*" or signpost*)	96934

Additional File 1: Detailed search stratehy for meta-ethnography

5	TS=((multidisciplin* or interdisciplin* or "multi* disciplin*" or "inter disciplin*" or multisector* or intersector* or "multi* sector*" or "inter sector*" or multiorgani?ation* or interorgani?ation* or "multi* organi?ation*" or inter organi?ation*) and (link* or liais* or collaborat* or partner* or engage* or leverag* or broker* or coordinat* or co-ordinat*)) OR TS=(((voluntary or volunteer* or charit* or faith* or church* or community or third or nongovernment* or non-government* or nonprofit* or non-profit* or "not for profit*") NEAR/2 (sector$ or agenc* or organi?ation$)) and (link* or liais* or collaborat* or partner* or engage* or leverag* or broker* or coordinat* or co-ordinat*))	36511
6	TS=("social determinant*" or "social need*" or "psychosocial need*") OR TS=((health or social or psychosocial or economic) NEAR/2 (equit* or inequit* or equal* or inequal* or depriv*)) OR TS=((social* NEAR/2 (interact* or isolat*)) or loneliness or lonely)	101793
7	#3 AND (#4 OR #5 OR #6)	1113
8	#2 OR #7	2360
9	TS=(ambulatory NEAR/3 (care or setting$ or facilit* or ward$ or department$ or service$)) OR TS=((general or family) NEAR/2 (practi* or physician$ or doctor$)) OR TS=("primary care" or "primary health care" or "primary healthcare") OR TS=(clinic$ OR visit$) OR TS=((health* or medical) NEAR/2 (center$ or centre$)) OR TI=community OR TS=(community NEAR/3 (service$ or care or health*))	414733
10	#8 AND #9	1231
11	#1 OR #10	2281
12	TI=qualitative or TS=(qualitative NEAR/2 (stud* or research or analysis)) OR TS=(interview* or "focus group*" or "mixed method*" or "multiple method*" or multimethod* or multi-method*) OR TS=("grounded theory" or ethnograph* or ipa or phenomenolog* or "thematic analysis" or "narrative analysis")	515207
13	#11 AND #12	797

Additional File 1: Detailed search stratehy for meta-ethnography

HMIC

	HMIC Health Management Information Consortium <1979 to July 2021>	
1	exercise referral systems/	3
2	linkworkers/	82
3	social prescri*.ti,ab.	85
4	(link? adj2 (worker? or practitioner? or staff or personnel or officer? or team?)).ti,ab.	124
5	((care or healthcare or community or outreach or patient?) adj5 navigator?).ti,ab.	25
6	(navigator? adj5 (program* or intervention* or service* or system?)).ti,ab.	14
7	(((care or healthcare or community or outreach or patient?) adj2 navigat*) and (worker? or practitioner? or staff or personnel or team?)).ti,ab.	15
8	((navigat* adj2 (program* or intervention* or service* or system?)) and (worker? or practitioner? or staff or personnel or team?)).ti,ab.	16
9	1 or 2 or 3 or 4 or 5 or 6 or 7 or 8	298
10	community workers/ or community health workers/	127
11	receptionists/	48
12	health service receptionists/ or general practice receptionists/	21
13	(receptionist? or reception staff or reception personnel).ti,ab.	261
14	(health* adj (assistant? or aide? or advisor? or adviser? or advocate? or co-ordinator? or coordinator? or connector? or officer? or facilitator? or liaison or broker? or coach* or promoter? or mobili?er?)).ti,ab.	715
15	(community adj (assistant? or aide? or advisor? or adviser? or advocate? or co-ordinator? or coordinator? or connector? or officer? or facilitator? or liaison or broker? or coach* or promoter? or mobili?er?)).ti,ab.	50
16	10 or 11 or 12 or 13 or 14 or 15	1156
17	exp primary care/ or community health services/ or family health services/ or primary care groups/ or primary care nurses/ or primary care teams/ or primary care trusts/ or primary health workers/	28992
18	general practitioners/ or family practitioners/ or general practice staff/	9668
19	(ambulatory adj3 (care or setting? or facilit* or ward? or department? or service?)).ti,ab.	529
20	((general or family) adj2 (practi* or physician? or doctor?)).ti,ab.	18366
21	(primary care or primary health care or primary healthcare).ti,ab.	19944
22	(clinic? or visit?).ti,ab.	10738
23	((health* or medical) adj2 (center? or centre?)).ti,ab.	3260
24	community.ti.	12774
25	(community adj3 (service? or care or health*)).ti,ab.	16528
26	17 or 18 or 19 or 20 or 21 or 22 or 23 or 24 or 25	72417
27	16 and 26	555

28	9 or 27	848
29	mixed methods research/ or qualitative research/	1324
30	qualitative analysis/	80
31	interviews/ or observation/ or interviewing/	1317
32	field work/ or focus groups/ or qualitative techniques/	742
33	qualitative.ti. or (qualitative adj2 (stud* or research or analysis)).ti,ab.	5650
34	(interview* or focus group* or mixed method* or multiple method* or multimethod* or multi-method*).ti,ab.	21069
35	(grounded theory or ethnograph* or ipa or phenomenolog* or thematic analysis or narrative analysis).ti,ab.	2766
36	29 or 30 or 31 or 32 or 33 or 34 or 35	24254
37	28 and 36	195

OpenGrey

All fields search	Hits	References selected
social prescribing	3	0
social prescriber	0	0
social prescribers	0	0
link worker	5	0
link workers	1	0
links worker	2	0
Links workers	2	0
link working	31	0
patient navigator	0	0
patient navigators	1	0
patient navigation	2	0
care navigator	0	0
care navigators	0	0
care navigation	0	0
