# Supplementary material for: A meta-ethnography of the factors that shape link workers’ experiences of social prescribing
Source: BMC Med. 2024 Jul 4;22:280. doi: 10.1186/s12916-024-03478-w (PMC11225255; doi:10.1186/s12916-024-03478-w)
Supplement: Supplementary file 2 — Additional file 2. Initial concept mapping (PDF) [file 12916_2024_3478_MOESM2_ESM.pdf]

Additional File 1: Initial mind-mapping and conceptual development

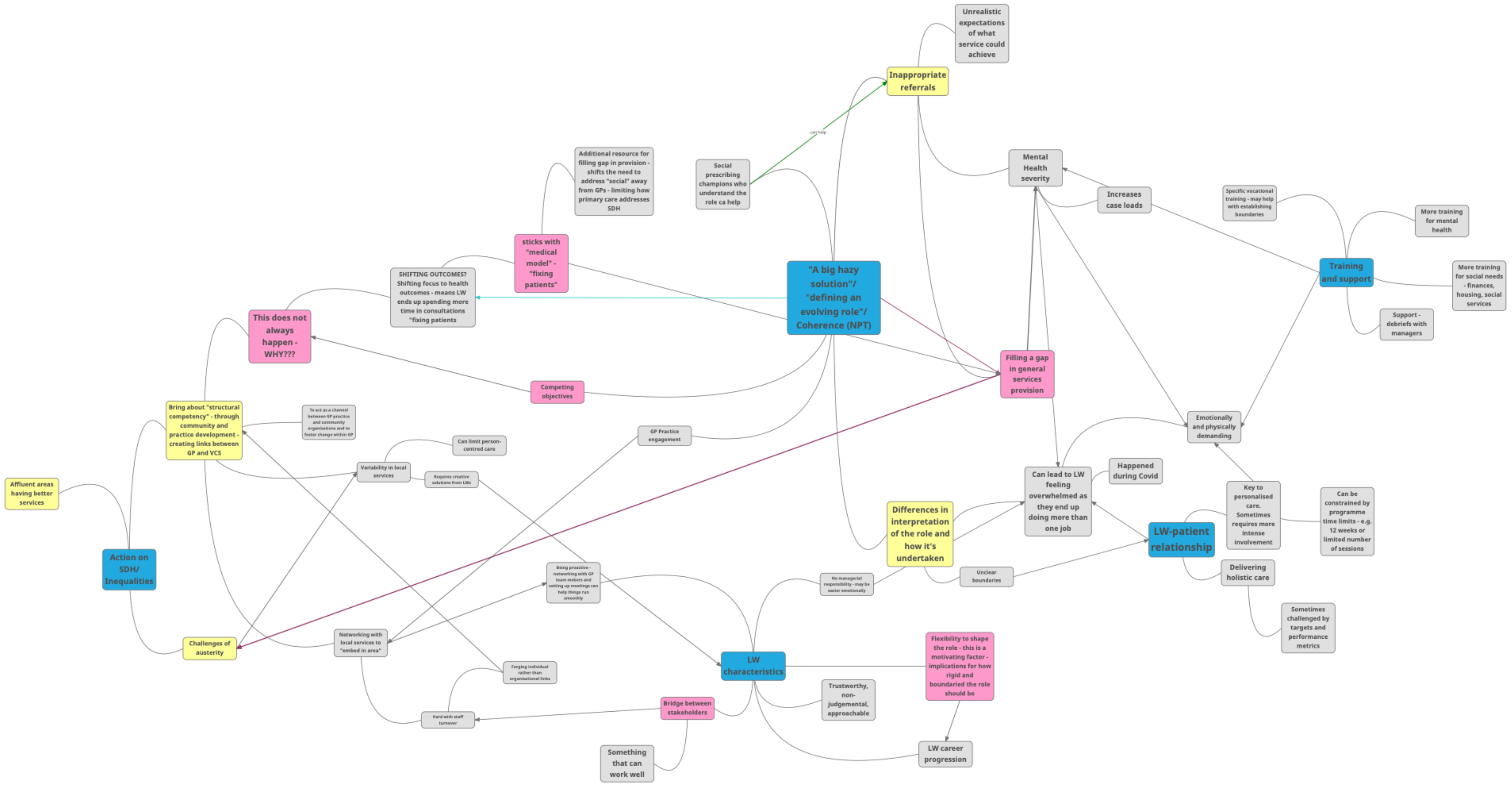

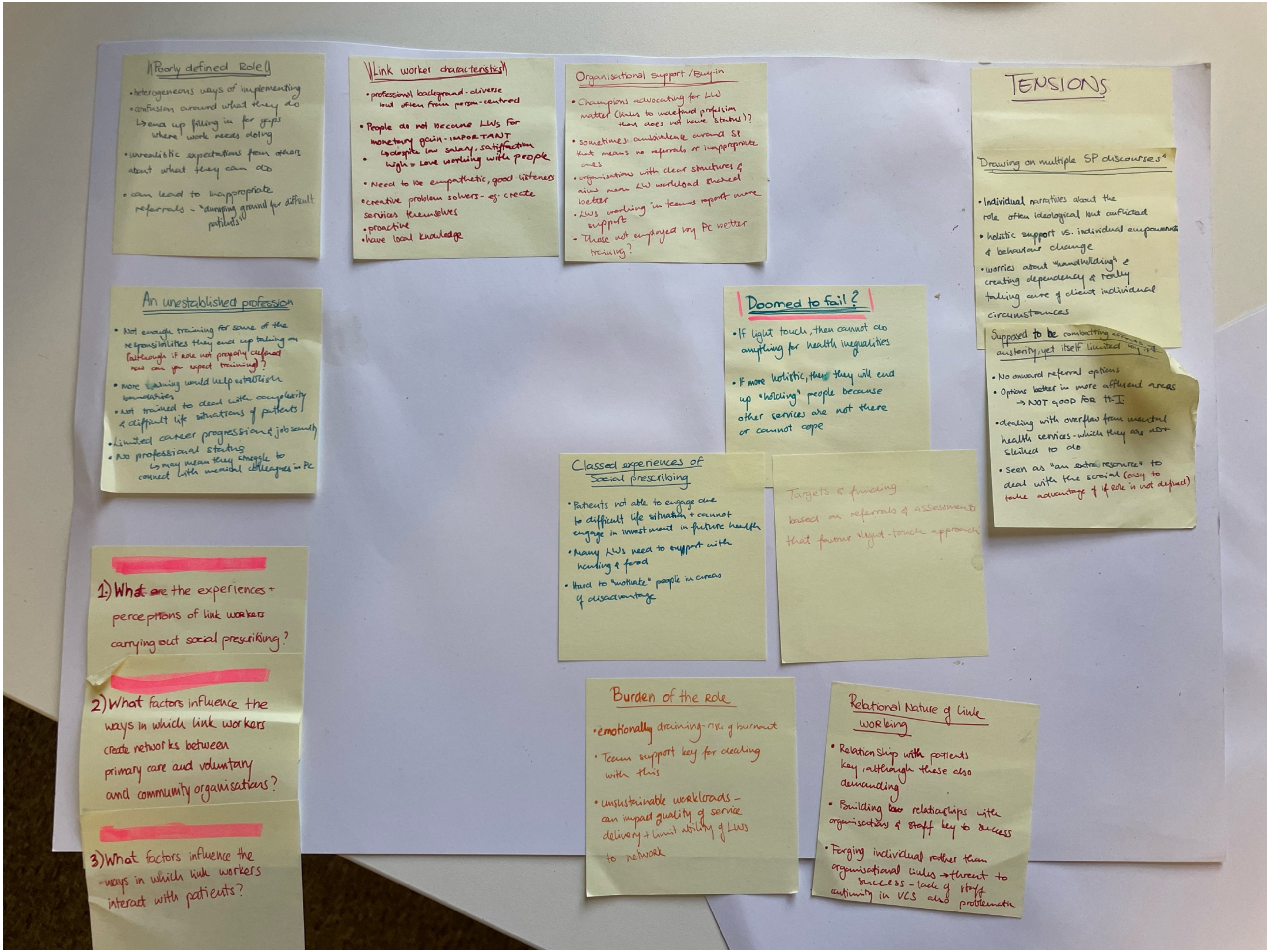
Poorly defined Role
• heterogeneous ways of implementing
• confusion around what they do
→ end up filling in for gaps where work needs doing
• unrealistic expectations from others about what they can do
• can lead to inappropriate referrals – "dumping ground for difficult patients"
Link worker characteristics
• professional background – diverse but often from person-centred
• People do not become LWs for monetary gain – IMPORTANT
→ despite low salary, satisfaction high = love working with people
• Need to be empathetic, good listeners
• creative problem solvers – eg. create services themselves
• proactive
• have local knowledge
Organisational support / Buy-in
• Champions advocating for LW matter (links to undefined profession that does not have status)?
• sometimes ambivalence around SP that means no referrals or inappropriate ones
• organisations with clear structures & aims mean LW workload shared better
• LWs working in teams report more support
- Those not employed by PC better training?
TENSIONS
"Drawing on multiple SP discourses"
• Individual narratives about the role often ideological but conflicted
• holistic support vs. individual empowerment & behaviour change
• worries about "handholding" & creating dependency & really taking care of client individual circumstances
Supposed to be combatting effects of austerity, yet itself limited by it
• No onward referral options
• Options better in more affluent areas
→ NOT GOOD FOR HI
• dealing with overflow from mental health services – which they are not skilled to do
• Seen as "an extra resource" to deal with the social (easy to take advantage of if role is not defined)
An unestablished profession
• Not enough training for some of the responsibilities they end up taking on (although if role not properly defined how can you expect training)?
• more training would help establish boundaries
• not trained to deal with complexity & difficult life situations of patients
• Limited career progression & job security
• No professional status
→ may mean they struggle to connect with medical colleagues in PC
Doomed to fail?
• If light touch, then cannot do anything for health inequalities
• If more holistic, then they will end up "holding" people because other services are not there or cannot cope
Classed experiences of Social prescribing
• Patients not able to engage due to difficult life situation + cannot engage in investment in future health
• Many LWs need to support with heating & food
• Hard to "motivate" people in areas of disadvantage
Targets & funding based on referrals & assessments that favour light-touch approach
1) What are the experiences + perceptions of link workers carrying out social prescribing?
2) What factors influence the ways in which link workers create networks between primary care and voluntary and community organisations?
3) What factors influence the ways in which link workers interact with patients?
Burden of the role
• emotionally draining – risk of burnout
• Team support key for dealing with this
• unsustainable workloads – can impact quality of service delivery + limit ability of LWs to network
Relational Nature of Link working
• Relationship with patients key, although these also demanding
• Building relationships with organisations & staff key to success
• Forging individual rather than organisational links → threat to success – lack of staff continuity in VCS also problematic
