# Supplementary material for: A meta-ethnography of the factors that shape link workers’ experiences of social prescribing
Source: BMC Med. 2024 Jul 4;22:280. doi: 10.1186/s12916-024-03478-w (PMC11225255; doi:10.1186/s12916-024-03478-w)
Supplement: Supplementary file 3 — Additional file 2. Critical Appraisal using the CASP tool (PDF) [file 12916_2024_3478_MOESM3_ESM.pdf]

Additional file 2: Critical Appraisal using the CASP tool

Reference	Section A: Are the results valid?						Section B: What are the results?		
	Clear aims	Is qualitative methodology appropriate?	Was the researchdesign appropriate to address the aims of theresearch?	Was the recruitment strategy appropriate to the aims of the research?	Was the data collected in a way that addressed the research issue?	Has the relationship between researcher and participants been adequately considered?	Have ethical issues been taken into consideration?	Was the data analysis sufficiently rigorous?	Is there a clear statement of findings?
Beardmore 2019	yes	yes	yes	yes	yes	no	can't tell	can't tell	yes
Chng et al. 2021	yes	yes	yes	can't tell	yes	no	yes	yes	yes
Fixsen et al. 2020	yes	yes	yes	yes	yes	can't tell	yes	yes	yes
Fixsen et al. 2021	yes	yes	yes	yes	yes	can't tell	yes	yes	yes
Frostick and Bertotti 2019	yes	yes	yes	yes	yes	yes	yes	yes	yes
Girffith et al.	yes	yes	yes	yes	yes	no	yes	can't tell	yes
Griffiths et al. 2023	can't tell	yes	yes	yes	yes	can't tell	yes	can't tell	yes
Hazeldine et al. 2020	yes	yes	yes	yes	yes	yes	yes	can't tell	yes
Holding et al. 2020	yes	yes	yes	yes	yes	can't tell	yes	yes	yes
Khan et al.	yes	yes	can't tell	yes	yes	can't tell	yes	can't tell	yes
Mackenzie et al.	yes	yes	yes	yes	yes	can't tell	yes	yes	yes
Mackenzie et al. 2020	yes	yes	yes	yes	yes	can't tell	yes	yes	yes
Moore et al. 2023	yes	yes	yes	yes	yes	can't tell	yes	can't tell	yes
Morris et al. 2022	yes	yes	yes	yes	yes	can't tell	yes	yes	yes
Pollard et al. 2023	yes	yes	yes	yes	yes	can't tell	yes	yes	yes
Rhodes and Bell 2021	yes	yes	yes	yes	yes	yes	yes	yes	yes
Simpson et al. 2020	yes	yes	yes	yes	yes	can't tell	yes	yes	yes
Skivington et al. 2019	yes	yes	yes	yes	yes	yes	yes	yes	yes
White et al. 2022	yes	yes	yes	yes	yes	yes	yes	yes	yes
Wildman, Moffatt, Penn, et al., 2019	yes	yes	yes	yes	yes	can't tell	yes	yes	yes
Wildman, Moffatt, Steer, et al., 2019	yes	yes	yes	yes	yes	can't tell	yes	yes	yes
